# Ellagic acid-Fe@BSA nanoparticles for endogenous H_2_S accelerated Fe(III)/Fe(II) conversion and photothermal synergistically enhanced chemodynamic therapy

**DOI:** 10.7150/thno.41882

**Published:** 2020-03-04

**Authors:** Qingqing Tian, Lu An, Qiwei Tian, Jiaomin Lin, Shiping Yang

**Affiliations:** The Key Laboratory of Resource Chemistry of the Ministry of Education, the Shanghai Key Laboratory of Rare Earth Functional Materials, and the Shanghai Municipal Education Committee Key Laboratory of Molecular Imaging Probes and Sensors, Shanghai Normal University, Shanghai, 200234 (China)

**Keywords:** endogenous H_2_S, Fe(III)/Fe(II) transformation, photothermal therapy, chemodynamic therapy, colon cancer treatment

## Abstract

**Rationale**: Chemodynamic therapy (CDT) based on the Fe(II)-mediated Fenton reaction is an emerging tumor treatment strategy. However, the catalytic efficiency in tumors is crucially limited by Fe(II). Herein, an endogenous hydrogen sulfide (H_2_S) accelerated Fe(III)/Fe(II) transformation and photothermal synergistically enhanced CDT strategy based on ellagic acid-Fe-bovine serum albumin (EA-Fe@BSA) nanoparticles (NPs) was developed for colon tumor inhibition. On the one hand, the Fe(III) with low catalytic activity in the EA-Fe@BSA NPs could be rapidly reduced to the highly active Fe(II) by the abundant H_2_S in colon cancer tissues. Thus, a rapid Fe(III)/Fe(II) conversion system was established, wherein highly active Fe(II) ions were continuously regenerated to improve the CDT efficiency. On the other hand, the photothermal effect of EA-Fe@BSA NPs also accelerated the production of hydroxyl radicals (•OH), thereby synergistically enhancing the CDT performance and improving the therapeutic efficacy.

**Methods**: The endogenous H_2_S accelerated Fe(III)/Fe(II) conversion and PTT enhanced CDT were investigated by characterization of the Fe valence state and detection of •OH. *T*_1_-weighted magnetic resonance imaging (MRI) was tested both *in vitro* and *in vivo*. The biocompatibility of NPs were examined via MTT assay, hemolysis analysis and routine blood measurements. The enhanced CDT was investigated in HCT116 colon cancer cells by Calcein-AM/PI staining and MTT assay, and tumor inhibition was demonstrated in HCT116 tumor bearing mice.

**Results**: In this work, EA-Fe@BSA NPs were constructed as a CDT theranostic reagent. The H_2_S accelerated Fe(III)/Fe(II) conversion was confirmed, more degradation of MB and generation of •OH demonstrated the enhanced CDT *in vitro*. EA-Fe@BSA NPs exhibited good *T*_1_-weighted MRI performance. More importantly, it displayed strong near-infrared (NIR) absorption and excellent photothermal efficiency, further promotes the production of •OH. Hence, the efficacy of CDT was enhanced, and the tumor growth was inhibited efficiently.

**Conclusion**: All results demonstrate that this strategy based on endogenous H_2_S promoted Fe(III)/Fe(II) transformation together with PTT acceleration permits efficient Fenton-reaction- mediated CDT both *in vitro* and* in vivo*, which holds great potential for effective colon cancer theranostics.

## Introduction

Colon cancer is one of the most common cancers worldwide. Its early symptoms are not obvious, but systemic symptoms such as anemia and weight loss occur in the terminal stage 1,2. Both its morbidity and mortality are very high. By far the most common clinical diagnosis and treatment strategies for colon cancer are colonoscopy, surgery, and radiation therapy. However, there are huge risks of recurrence and metastasis, accompanied by great pain. Hence, it is imperative to develop more effective minimally invasive approaches for diagnosing and treating colon cancer. Interestingly, it is well known that the enzyme cystathionine-β-synthase (CBS) can produce hydrogen sulfide (H_2_S) and is selectively up-regulates in colon cancer cells, thus, the concentration of H_2_S in colon cancer tissues (0.3-3.4 mmol L^-1^) is considerably higher than that in non-tumor tissues 3-7. Taking advantage of this property, in our previous work, we designed Cu_2_O nanoparticles (NPs) for colon cancer theranostics, which could react with H_2_S to afford CuS with high absorption in the near-infrared (NIR) region for photothermal therapy (PTT). Nevertheless, the concentrations of Cu_2_O and H_2_S required for good treatment effects were both extremely high, which hindered its further application 8. Thus, it is crucial to explore more effective approaches and develop novel intelligent agents for the treatment of colon cancer.

An emerging strategy in recent years is chemodynamic therapy (CDT), in which endogenous H_2_O_2_ is converted to hydroxyl radicals (•OH) via metal-ion-catalyzed Fenton or Fenton-like reactions at the tumor site 9. The •OH (*E*(•OH/H_2_O) = 2.80 V) is highly toxic and a more oxidizing reactive oxygen species (ROS) than H_2_O_2_ (*E*(H_2_O_2_/H_2_O) = 1.78 V), therefore causing harmful oxidative damage to tumors 10-15. The efficiency of CDT is dependent on the H_2_O_2_ concentration, pH, and metal catalyst, and substantial efforts have been devoted to designing CDT nanotheranostic agents with high catalytic performance, in which the Fe(II)-initiated Fenton reaction has been the most widely applied. In the Fe(II)-catalyzed system, Fe(II) catalyzes the conversion of H_2_O_2_ to •OH and is itself converted to Fe(III), which subsequently mediate Fe(III)-catalyzed Fenton-like reactions with lower catalytic activity. Meanwhile, the rate of Fe(III)/Fe(II) transformation is slow (0.002-0.01 M^-1^ s^-1^), which greatly limits the rate of the Fenton reaction and thus hinders the further application of CDT 16-22. To date, several strategies have been proposed for enhancing CDT performance, including thermal acceleration, PTT enhancement, valence state conversion, and self-supplied H_2_O_2_ 23-30. For Fe(III)/Fe(II) conversion, one of the most common approaches is introducing a reducing agent or semiconductor to Fe materials to catalyze the regeneration of Fe(II), but this strategy often requires light excitation, additional reagents, or complex protocols 31-33. Thus, it is pressing to propose a facile method for Fe(III)/Fe(II) rapid conversion. Another strategy to enhance CDT performance is increasing the temperature at the tumor site. PTT, in which the absorbed NIR laser energy generates local thermal effects for tumor treatment, has attracted considerable attention owing to its low cost, minimal invasiveness, and tumor specificity 34-37. Importantly, the heating effect of PTT could accelerate the production of •OH, thus enhancing the CDT efficiency and providing more effective treatment 38-41. Therefore, the development of a CDT reagent with high catalytic activity based on Fe(III)/Fe(II) conversion and PTT would be of great value.

**Scheme 1:**
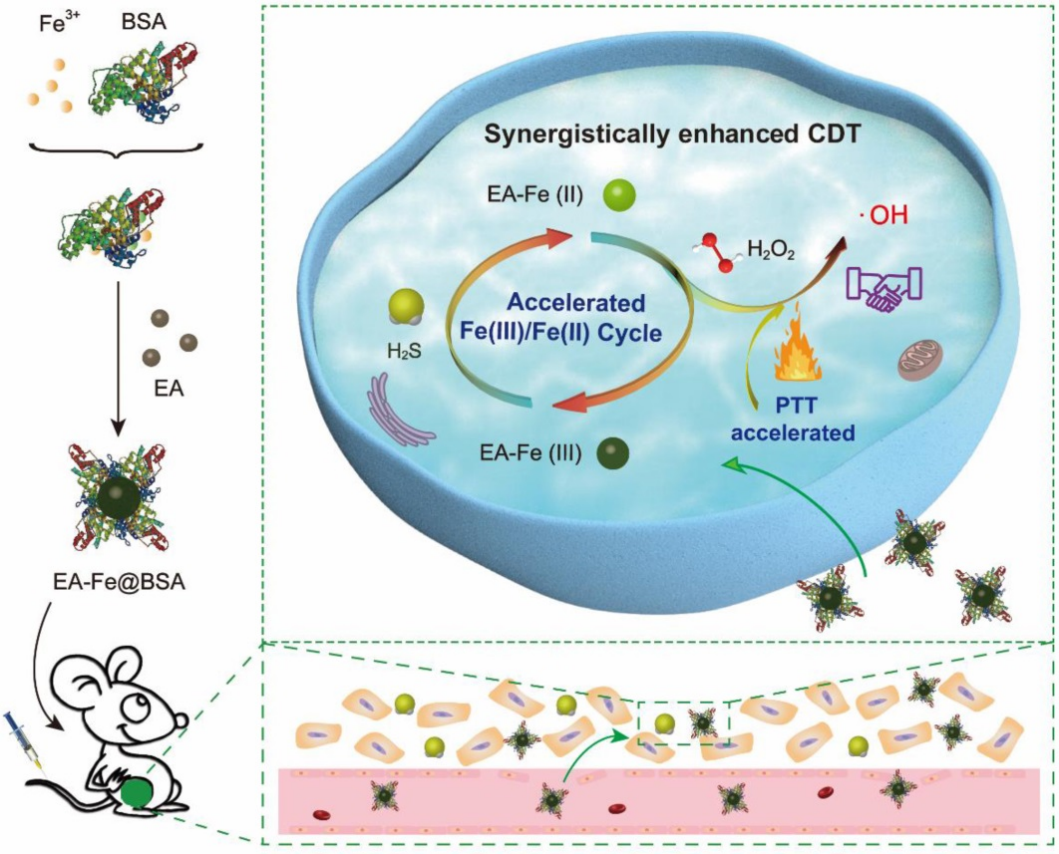
Schematic illustration of the preparation of the EA-Fe@BSA NPs and endogenous H_2_S accelerated Fe(III)/Fe(II) conversion and PTT synergistically enhanced CDT.

As a proof of concept, ultra-small ellagic acid-Fe-bovine serum albumin (EA-Fe@BSA) NPs were constructed as a CDT reagent to demonstrate the Fe(III)/Fe(II) conversion and PTT enhanced CDT. The EA-Fe@BSA NPs with good biocompatibility was prepared via a simple assembly of natural polyphenol, Fe(III) and albumin for endogenous H_2_S and PTT accelerated CDT efficacy (Scheme 1). The key feature of this approach is acceleration of the Fe(III)/Fe(II) transformation to regenerate highly active Fe(II) via the strong reducing ability of endogenous H_2_S in colon cancer tissues. Following the intravenous injection of the EA-Fe@BSA NPs into mice and their internalization by colon cancer tumors, the Fe(III) with low catalytic activity in the synthesized EA-Fe@BSA NPs was rapidly reduced to the active Fe(II) by the abundant endogenous H_2_S, thereby providing a rapid Fe(III)/Fe(II) transformation system for improved CDT. Furthermore, the obtained EA-Fe@BSA NPs exhibited strong NIR absorption and excellent thermal effects, thereby synergistically improving the CDT efficiency. Moreover, the EA-Fe@BSA NPs showed obvious *T*_1_-weighted magnetic resonance imaging (MRI) both *in vitro* and *in vivo*, demonstrating their potential application in colon tumor diagnosis and treatment. To the best of our knowledge, this is the first time that endogenous H_2_S has been utilized as a reducing agent to promote CDT efficacy for colon cancer treatment. Therefore, the developed strategy is expected to prove valuable for colon cancer treatment, which was confirmed by prominent tumor inhibition in HCT116 colon cancer tumor-bearing mice.

## Results and discussion

### Synthesis and Characterization of EA-Fe@BSA NPs

**Figure 1:**
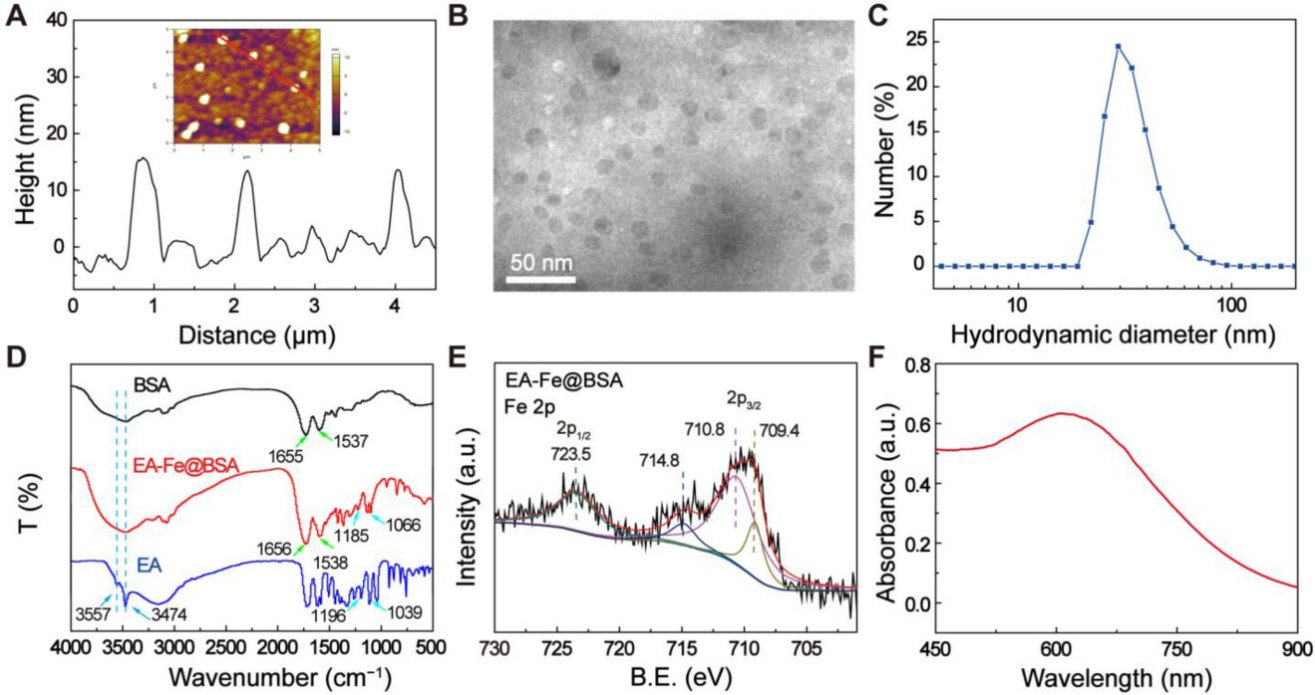
Characterization of the EA-Fe@BSA NPs: (A) 3D AFM height profile, (B) TEM image, (C) hydrodynamic diameter profile, (D) FT-IR spectra of BSA, EA, and the EA-Fe@BSA NPs, (E) XPS spectra and fitted curves, (F) absorbance spectrum.

The EA-Fe@BSA NPs were synthesized at room temperature as follows. Aqueous FeCl_3_ solution was added to BSA solution, followed by the addition of EA under stirring to form a dark blue solution. The EA-Fe@BSA NPs were isolated by ultrafiltration centrifugation. The size and morphology of the as-synthesized NPs were characterized via atomic force microscopy (AFM) (Figure 1A). The prepared NPs exhibited a monodisperse spherical morphology with an average diameter of 14.41 ± 0.08 nm (Figure S1). Transmission electron microscopy (TEM) further confirmed that the EA-Fe@BSA NPs (Figure 1B) were monodisperse spheres with a diameter of 13.84 ± 2.53 nm (Figure S2), in accordance with the AFM results. In addition, the EA-Fe@BSA NPs displayed an average hydrodynamic diameter of approximately 29.2 nm (Figure 1C). The hydrodynamic diameter was slightly larger than the diameter measured by AFM and TEM, which was attributed to the hydration shells and strongly hydrophilic BSA. More importantly, the EA-Fe@BSA NPs exhibit excellent dispersibility and stability in different medium including water, PBS and plasma (Figure S3 and S4).

The composition, structural characteristics, and valence of the EA-Fe@BSA NPs were investigated via Fourier-transform infrared (FT-IR) spectroscopy, X-ray photoelectron spectroscopy (XPS), and UV-vis absorption spectroscopy. The bands at 1656 cm^-1^ (amide I band vibrations) and 1538 cm^-1^ (amide II band vibrations) observed in the FT-IR spectrum of the EA-Fe@BSA NPs confirmed the presence of BSA. In addition, the slight shift relative to pure BSA of FT-IR spectrum and the fluorescence quenching observed in the fluorescence spectrum further indicated the coordination between Fe and BSA (Figure 1D and S5). In addition, the EA-Fe@BSA NPs exhibited characteristic bands (1185 and 1066 cm^-1^) similar to those of pure EA (1196 and 1039 cm^-1^) corresponding to ester C-O stretching, demonstrating the presence of EA. However, the infrared bands displayed a slight shift, indicating the interaction of the EA with the Fe ions. Moreover, the OH stretching bands of pure EA at 3557 and 3474 cm^-1^ were not observed for the EA-Fe@BSA NPs, demonstrating the successful chelation of the Fe ions by the catechol moieties of EA in the EA-Fe@BSA NPs 42. The valence state of the Fe in the EA-Fe@BSA NPs was further analyzed via XPS. As shown in Figure 1E and S6, peaks corresponding to Fe 2p_1/2_ (723.5 eV) and Fe 2p_3/2_ (709.4, 710.8, and 714.8 eV) were observed. The peak at 709.4 eV could be attributed to Fe(II), whereas those at 710.8 and 714.8 eV could be attributed to Fe(III); the peak area ratio of Fe(III)/Fe(II) is about 5.5: 1. These results indicate that the Fe in the EA-Fe@BSA NPs existed in a mixed valence state consisting of both Fe(III) and Fe(II), where Fe(III) was the predominant species 43,44. In addition, the EA-Fe@BSA NPs exhibited strong absorption throughout the visible and NIR region compared with EA and BSA alone (Figure S7), and the characteristic absorption peak at 609 nm may be attributable to the d-d electronic transition of the EA-Fe complex (Figure 1F) 45. These results indicated the potential of the NPs as a photothermal agent for cancer treatment. All of the above results indicated the successful synthesis of the EA-Fe@BSA NPs.

### Enhanced CDT Performance *In Vitro*

**Figure 2:**
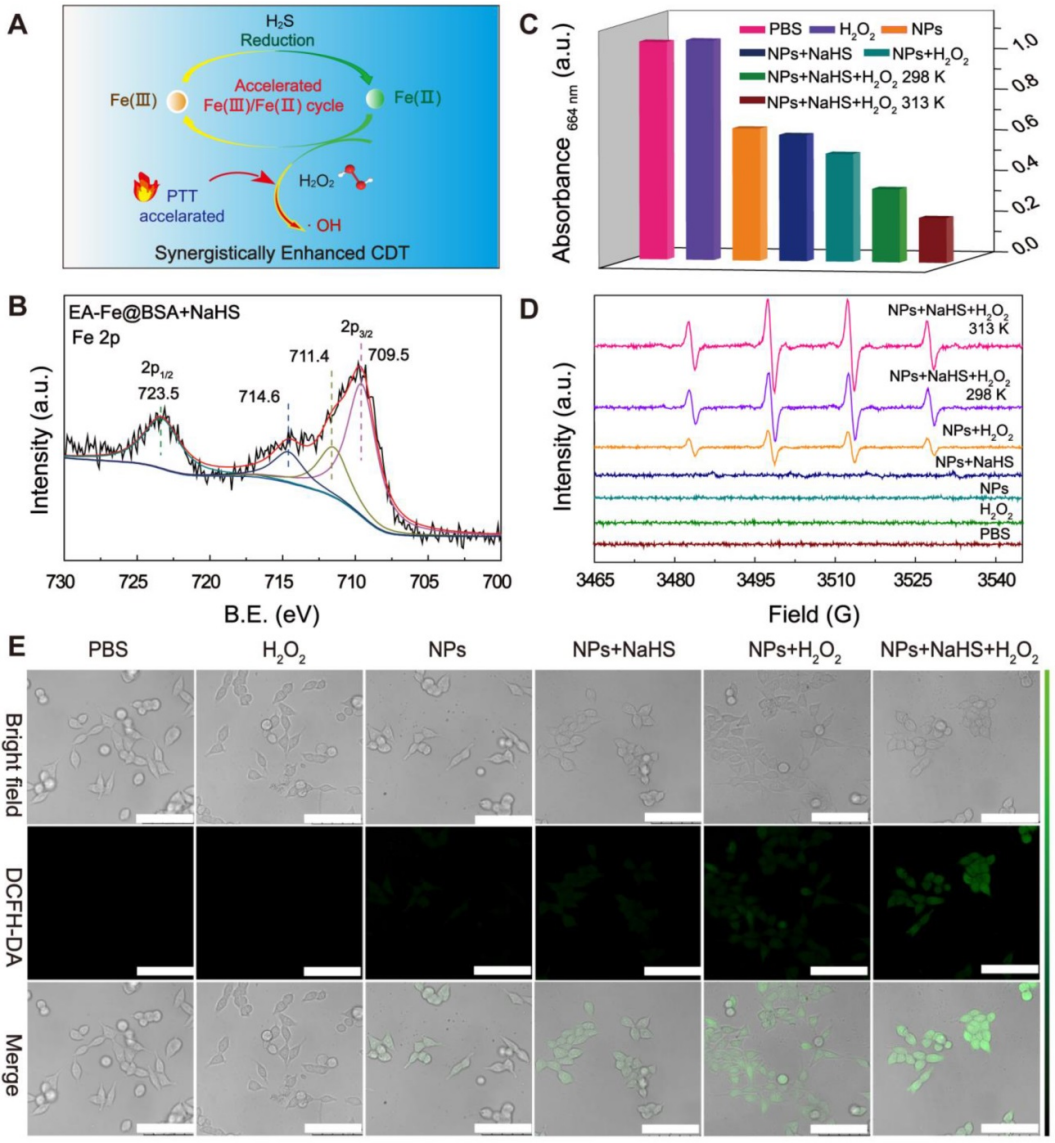
Enhanced CDT performance *in vitro*. (A) Mechanism of H_2_S accelerated Fe(III)/Fe(II) transformation to enhance CDT. (B) XPS spectra and fitted curves of EA-Fe@BSA NPs after NaHS addition. (C) MB absorbance values at 664 nm under different conditions, indicating the production of •OH. (D) ESR spectra for •OH detection. (E). Confocal laser scanning microscopy images of HCT116 colon cancer cells stained with DCFH-DA under various conditions (A_1_: PBS, A_2_: H_2_O_2_, A_3_: NPs, A_4_: NPs + NaHS, A_5_: NPs + H_2_O_2_, A_6_: NPs + NaHS + H_2_O_2_ at 298 K, and A_7_: NPs + NaHS + H_2_O_2_ at 313 K; scale bars = 75 μm).

The proposed mechanistic process of Fe(III)/Fe(II) conversion in the EA-Fe@BSA NPs is as follows. After entering the H_2_S-rich colon cancer tumor environment, the Fe(III) with low catalytic activity were rapidly reduced by H_2_S to the more active Fe(II), thereby realizing a rapid Fe(III)/Fe(II) transformation system for improved CDT performance. Moreover, the thermal effect led to the generation of more •OH and further improved the CDT performance (Figure 2A). Sodium hydrosulfide (NaHS) was selected to simulate endogenous H_2_S to investigate the H_2_S-enhanced CDT performance *in vitro*. The valence state of the Fe after the addition of NaHS to the EA-Fe@BSA NPs was analyzed via XPS. As shown in Figure 2B, peaks corresponding to Fe 2p_1/2_ (723.5 eV) and Fe 2p_3/2_ (709.5, 711.4, and 714.6 eV) appeared. The peak at 709.5 eV could be attributed to Fe(II), whereas those at 711.4 and 714.6 eV could be ascribed to Fe(III). The peak areas clearly indicated that Fe(II) was the more dominant species, the peak area ratio of Fe(III)/Fe(II) is about 0.5: 1, indicating the reduction of Fe(III) to Fe(II) by NaHS 46,47. To further confirm the conversion of Fe(III) to Fe(II), 1,10-phenanthroline, which is commonly used as an indicator of Fe(II), was added and the absorbance was detected at 511 nm. As shown in Figure S8, NaHS solution was added to solutions of FeCl_3_ or EA-Fe@BSA, respectively, followed by the addition of 1,10-phenanthroline. The addition of NaHS led to a significant increase in the absorption peak at 511 nm, indicating the reduction of Fe(III) to Fe(II) by NaHS, which is similar to that of the other reductive substance, such as GSH (Figure S9) 48. More importantly, the EA-Fe@BSA NPs still exhibit good stability after adding the NaHS (Figure S10). Subsequently, methylene blue (MB) was used to evaluate the generation of •OH. Seven experiments were performed under different conditions (A_1_: PBS, A_2_: H_2_O_2_, A_3_: NPs, A_4_: NPs + NaHS, A_5_: NPs + H_2_O_2_, A_6_: NPs + NaHS + H_2_O_2_ at 298 K, and A_7_: NPs + NaHS + H_2_O_2_ at 313 K), in which these reagents were added to the same concentration of MB solution and reacted for 30 min prior to measuring the absorbance of the solution after centrifugation (Figure S11). The A_1_ and A_2_ samples were used as controls. As illustrated in Figure 2C, the MB absorption peak at 664 nm was reduced by approximately 39% in the A_3_ and A_4_ samples, which was attributed to adsorption of the MB on the surface of the EA-Fe@BSA NPs. The MB absorbance was reduced by approximately 49% in the A_5_ sample, which was ascribed to the production of •OH by the EA-Fe@BSA NPs in the presence of H_2_O_2_. The MB absorbance was reduced by approximately 66% in the A_6_ sample owing to reduction of the Fe(III) with low catalytic activity in the EA-Fe@BSA NPs to high active Fe(II) by NaHS. It is worth noting that the MB absorbance was reduced by approximately 80% in the A_7_ sample, which was ascribed to a combination of the reduction of the Fe(III) in the EA-Fe@BSA NPs to Fe(II) by NaHS and the accelerated production of •OH due to the higher temperature. In the conventional Fenton reaction, Fe(II) is oxidized to Fe(III) with the concomitant generation of •OH from H_2_O_2_, while the rate of Fe(III)/Fe(II) transformation is very slow (0.002-0.01 M^-1^ s^-1^), which limits the efficiency of CDT 20. The addition of NaHS and increased temperature solved this problem well, facilitating the Fe(III)/Fe(II) transformation and thereby accelerating the CDT.

To further explore the CDT performance of the EA-Fe@BSA NPs, the generation of •OH was examined by electron spin resonance (ESR) under seven different sets of conditions. 5,5-Dimethyl-1-pyrroline *N*-oxide (DMPO), which could capture •OH and exhibit a characteristic four-line 1:2:2:1 spectrum in ESR, was used as a capture agent. As shown in Figure 2D, the characteristic four-line pattern was not observed in the spectra of the A_1_-A_4_ samples. In contrast, the four-line pattern was clearly observed in the presence of both EA-Fe@BSA NPs and H_2_O_2_ (A_5_ sample), as the NPs reacted with H_2_O_2_ to produce •OH. As expected, a stronger pattern was observed in the presence of NPs, NaHS, and H_2_O_2_ (A_6_ sample), in accordance with the results of the MB discoloration experiment, confirming that NaHS accelerated the transformation of Fe(III)/Fe(II) and therefore the production of •OH. To our delight, upon increasing the temperature (A_7_ sample), more •OH was detected, further demonstrating the acceleration of the Fenton reaction due to thermal effects and providing the foundation for the subsequent PTT enhancement of CDT.

To evaluate the enhanced CDT performance at the cellular level, 2ʹ,7ʹ-dichlorodihydrofluorescein diacetate (DCFH-DA), which dyes ROS to afford green fluorescence, was used as a staining agent to reveal the production of •OH in the presence of H_2_O_2_. As illustrated in Figure 2E, no green fluorescence was observed in the A_1_ and A_2_ samples, while only weak fluorescence was observed in the A_3_ and A_4_ samples owing to the presence of a small amount of H_2_O_2_ in the interstitial cells that reacted with the NPs to generate •OH. This fluorescence was slightly stronger in the A_4_ sample than in the A_3_ sample, which was attributed to the NaHS-mediated reduction of the Fe(III)) in the EA-Fe@BSA NPs to Fe(II). In the A_5_ and A_6_ samples, the fluorescence was significantly enhanced compared with the samples lacking H_2_O_2_, because the addition of exogenous H_2_O_2_ increased the Fenton reaction efficacy. Furthermore, the A_6_ sample exhibited the strongest fluorescence, which was consistent with the results of the MB decolorization experiment and ESR spectra. Owing to a large amount of •OH generated by the NaHS and thermal effects, the cells were almost completely killed in the A_7_ sample and could not be photographed by confocal laser scanning microscope. All of these results demonstrate that the Fe(III) with low catalytic activity was rapidly reduced by NaHS to the highly active Fe(II) species, resulting in a rapid Fe(III)/Fe(II) transformation system for improving the Fenton reaction, combined with thermally enhanced CDT to generate a large amount of •OH, indicating promising performance as an enhanced CDT system for treating colon cancer* in vivo*.

### Photothermal and MRI Performance

**Figure 3:**
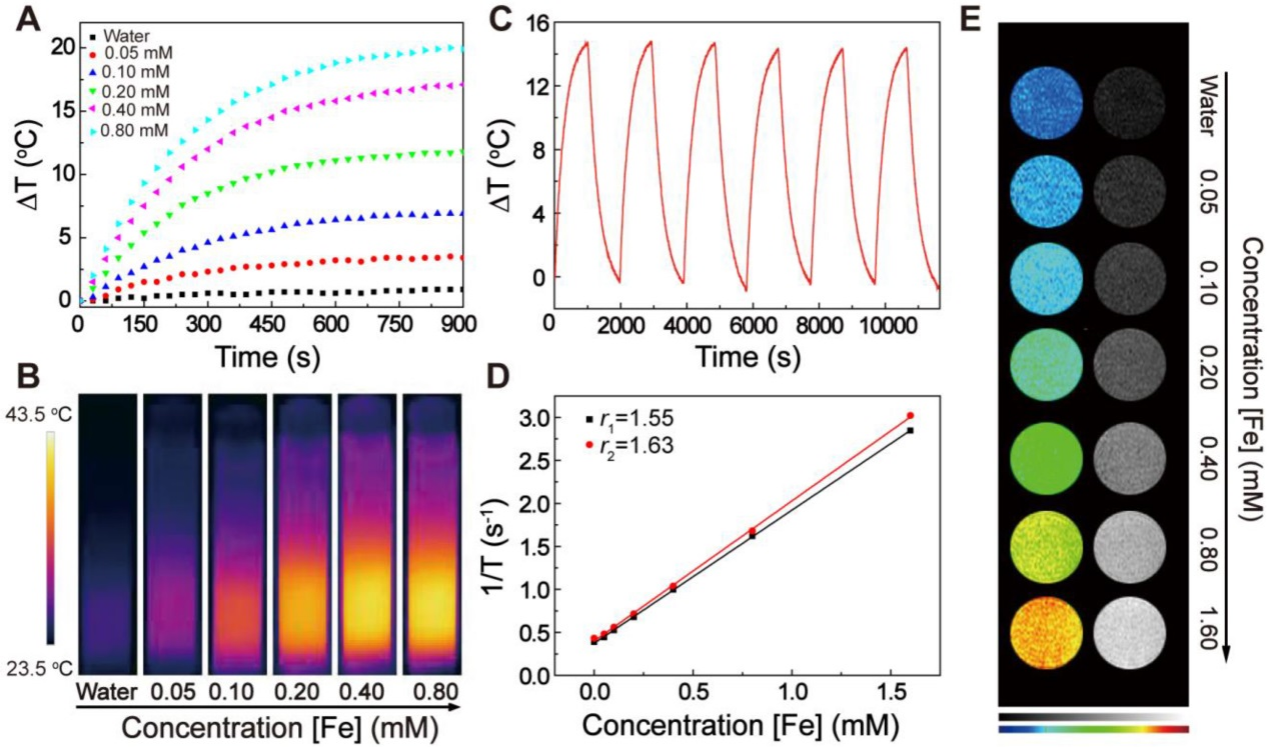
Characterization of the photothermal and MRI performance of the EA-Fe@BSA NPs. (A) Photothermal heating curves of the EA-Fe@BSA NPs at various Fe concentrations (0, 0.05, 0.10, 0.20, 0.40, and 0.80 mM) under irradiation with an 808 nm laser (1 W/cm^2^). (B) NIR thermal images corresponding to (A). (C) Photothermal cycle measurement for the EA-Fe@BSA NPs. (D) Relaxation rates (*r*_1_ and* r*_2_) of solutions of the EA-Fe@BSA NPs at 0.5 T. (E) *T*_1_-weighted images corresponding to (D).

The photothermal properties of the EA-Fe@BSA NPs resulting from their strong NIR absorption were examined. Solutions containing various Fe concentrations of the NPs (0, 0.05, 0.10, 0.20, 0.40, or 0.80 mM) were irradiated with an 808 nm (1 W/cm^2^) laser for 15 min and the temperature was monitored. As shown in Figure 3A, the solution temperature increased with increasing Fe concentration of the NPs, indicating a concentration-dependent photothermal effect. The corresponding thermal images also revealed that the thermal effect of the EA-Fe@BSA NPs increased with increasing concentration (Figure 3B), clearly demonstrating that the EA-Fe@BSA NPs possess excellent photothermal properties and can rapidly convert NIR light into thermal energy. The temperate change of the EA-Fe@BSA NPs under the irradiation of the laser with different power density further demonstrated that the 1 W/cm^2^ is the better choose for therapy in our system (Figure S12). Furthermore, the NPs still displayed a good heating effect and morphology after six cycles of heating and cooling, confirming their good photothermal stability (Figure 3C and S13). The photothermal conversion efficiency of the EA-Fe@BSA NPs before and after reaction with NaHS was calculated to be approximately 31.9% and 31.2% (Figure S14), which is similar to previously reported values for Fe-polyphenol-based PTT agents 42. In addition, to examine the influence of H_2_S, the photothermal properties of the NPs after the addition of NaHS as an exogenous H_2_S source were also measured under the same conditions. No significant changes in the photothermal curves or NIR thermal images were observed (Figure S15 and S16), demonstrating the good stability of the EA-Fe@BSA NPs as a PTT agent for cancer treatment.

Fe-polyphenol complexes have also been reported to display good MRI performance 49, indicating the possible additional application of the EA-Fe@BSA NPs as an MRI contrast agent. The longitudinal (*T*_1_) and transverse (*T*_2_) relaxation times were measured for various Fe concentrations of NPs (0, 0.05, 0.10, 0.20, 0.40, 0.80, or 1.60 mM) and the relaxation rate of the NPs was calculated. As shown in Figure 3D, the longitudinal relaxation rate *r*_1_ was 1.55 mM^-1^ s^-1^ and the transverse relaxation rate *r*_2_ was 1.63 mM^-1^ s^-1^ 50, and consequently *r*_2_/*r*_1_ = 1.05 < 3, indicating that the EA-Fe@BSA NPs could serve as a *T*_1_-weighted contrast agent. *T*_1_-weighted MRI measurements confirmed this possibility. The imaging brightness clearly increased with increasing concentration of EA-Fe@BSA NPs, and the color of the *T*_1_-weighted images ranged from dark blue (pure water) to orange (Figure 3E). On the basis of these results, the EA-Fe@BSA NPs not only exhibit enhanced CDT effects but also act as a good photothermal agent and a *T*_1_-weighted MRI contrast agent for MRI-guided cancer treatment.

### Biocompatibility

**Figure 4:**
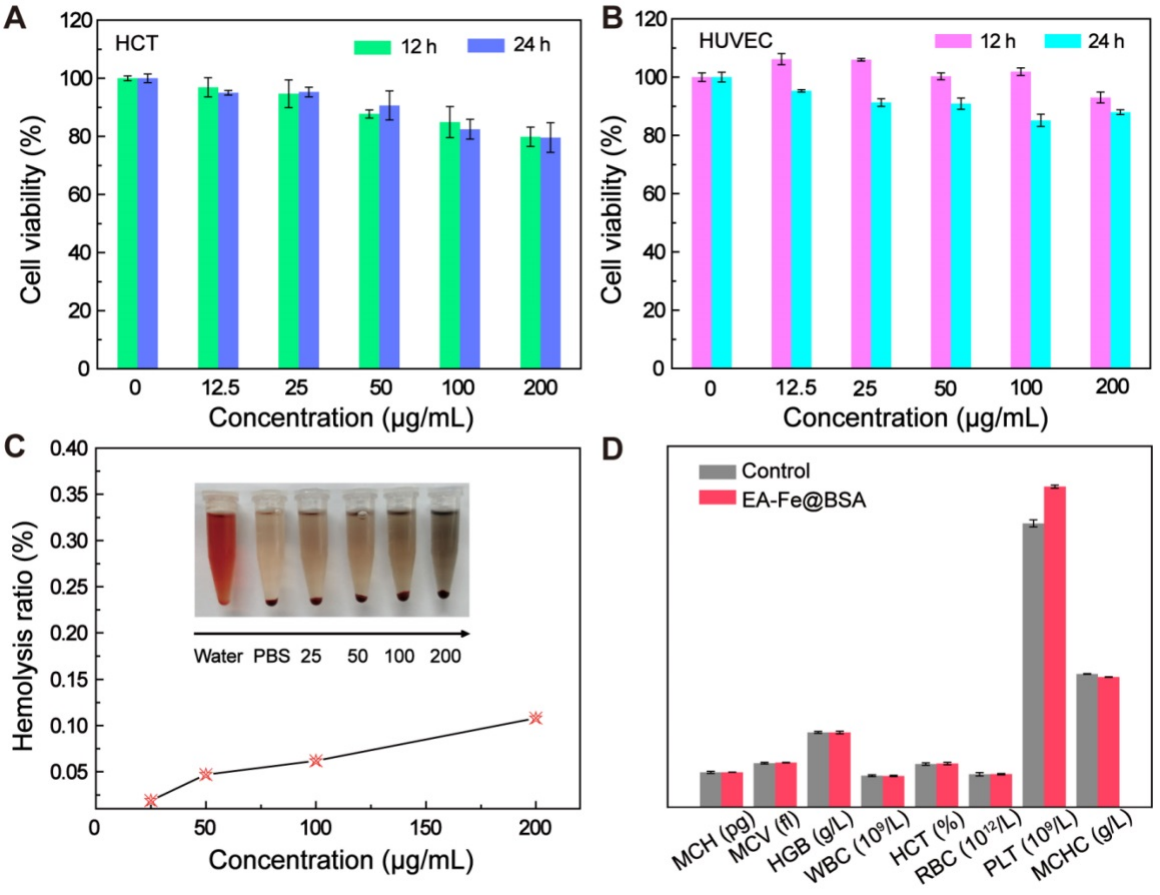
Biocompatibility of the EA-Fe@BSA NPs. Cell viability tests toward (A) HCT116 cells and (B) HUVECs after incubation with various concentrations of the EA-Fe@BSA NPs for 12 and 24 h. (C) Hemolysis assays for PBS, water, and various concentrations of the EA-Fe@BSA NPs. (D) Routine blood tests for normal mice and mice treated with the EA-Fe@BSA NPs (*n* = 3).

Good biocompatibility is essential for the biological application of the EA-Fe@BSA NPs *in vivo*. Therefore, the cytotoxicity of the NPs toward HCT116 cells and human umbilical vein endothelial cells (HUVECs), the hemolysis of red blood cells (RBCs), and routine blood measurements were used to evaluate the biocompatibility prior to *in vivo* experiments. Various concentrations of the EA-Fe@BSA NPs (0, 12.5, 50.0, 100.0, or 200.0 μg mL^-1^) were added to colon cancer cells and incubated for 12 or 24 h, after which the viability of the cells was counted using a microplate reader. As shown in Figure 4A, the cell viability decreased with increasing NPs concentration, but the survival rate remained above 80%. The toxicity of the NPs toward HUVECs was studied in a similar manner, and the cell survival rate was also in excess of 80%, indicating the good biocompatibility of the EA-Fe@BSA NPs (Figure 4B). A hemolysis assay was also performed for RBCs incubated with the EA-Fe@BSA NPs. The photograph of the blood supernatant dissolved in water was dark red (positive control), whereas transparent in PBS (negative control). No major hemolysis was observed even when the maximum concentration of the EA-Fe@BSA NPs was dispersed in PBS solution. The hemolysis ratio increased slightly with increasing NP concentration (Figure 4C and S17) but remained less than 5% even at the maximum concentration, further demonstrating the good biocompatibility of the EA-Fe@BSA NPs and establishing a robust foundation for subsequent biological applications. To investigate whether the EA-Fe@BSA NPs had effects on other tissues *in vivo*, routine blood tests were performed after intravenous injection of the NPs. Three normal nude mice were taken as the control group, and routine blood indices, including the red blood cell count (RBC, 10^12^/L), white blood cell count (WBC, 10^9^/L), average red blood cell hemoglobin (MCH, pg), mean red blood cell volume (MCV, fl), hemoglobin (HGB, g/L), red blood cell specific volume (HCT, %), platelet count (PLT, 10^9^/L), and mean corpuscular hemoglobin concentration (MCHC, g/L) were measured. As illustrated in Figure 4D, no significant difference was observed between the control and treated groups for most of the indices. The PLT of the NPs group was higher than that of the normal group, but it was still within the normal range 26. respectively, which are both within the normal range. From the above results, it can be concluded that the EA-Fe@BSA NPs displayed good biocompatibility and low toxicity and were suitable for *in vivo* experiments.

### *In Vivo* MRI

**Figure 5:**
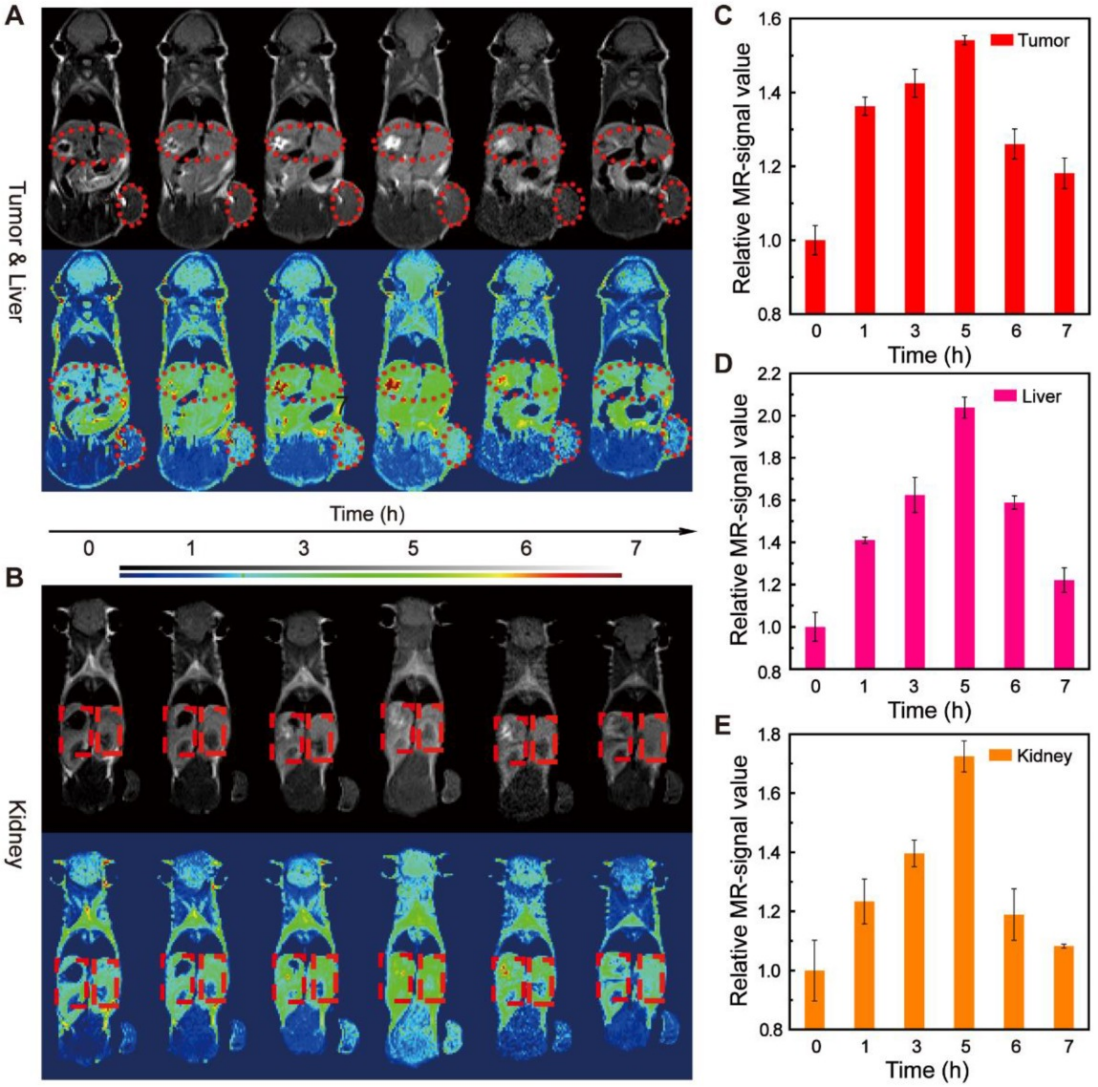
MRI images of the EA-Fe@BSA NPs *in vivo*. (A) MRI images of the tumor (indicated with small ellipses) and liver (indicated with large ellipses). (B) MRI images of the kidney (indicated with rectangles). (C-E) Corresponding MRI signal intensities for the images shown in (A, B).

Inspired by the good *T*_1_-weighted MRI results *in vitro*, we further explored the imaging performance of the EA-Fe@BSA NPs* in vivo*. Background images of colon cancer bearing mice were first acquired, and then the mice were intravenously injected with 3.5 mM Fe concentrations of NPs (20 mg/kg body weight) and images were collected at 1, 3, 5, 6, and 7 h after injection. As shown in Figure 5A, the tumor site became brighter and then darkened over time, and the brightest signal was observed after 5 h. The increased signal was ascribed to the accumulation of the EA-Fe@BSA NPs at the tumor site, where the NPs reached a maximum concentration after 5 h further demonstrate by the biodistribution of EA-Fe@BSA NPs in main organs (Figure S18). Subsequently, the NPs were metabolized and the signal at the tumor site became weaker. Similarly, the signals from the liver and kidneys first became brighter and then gradually darkened over time, and the brightest signal was again observed after 5 h (Figure 5A and 5B). The MRI signal intensity statistics for the tumor site were consistent with the imaging results, indicating that the EA-Fe@BSA NPs had good *T*_1_-weighted imaging performance for tumors (Figure 5C). The relative MRI signal intensities for the liver and kidneys exhibited a consistent trend over time (Figure 5D and 5E), revealing that the NPs underwent some degree of retention in the liver and kidneys. These results suggest that the EA-Fe@BSA NPs can be used as an effective *T*_1_-weighted MRI contrast agent for tumor-specific diagnosis.

### Enhanced CDT Therapy *In Vivo*

**Figure 6:**
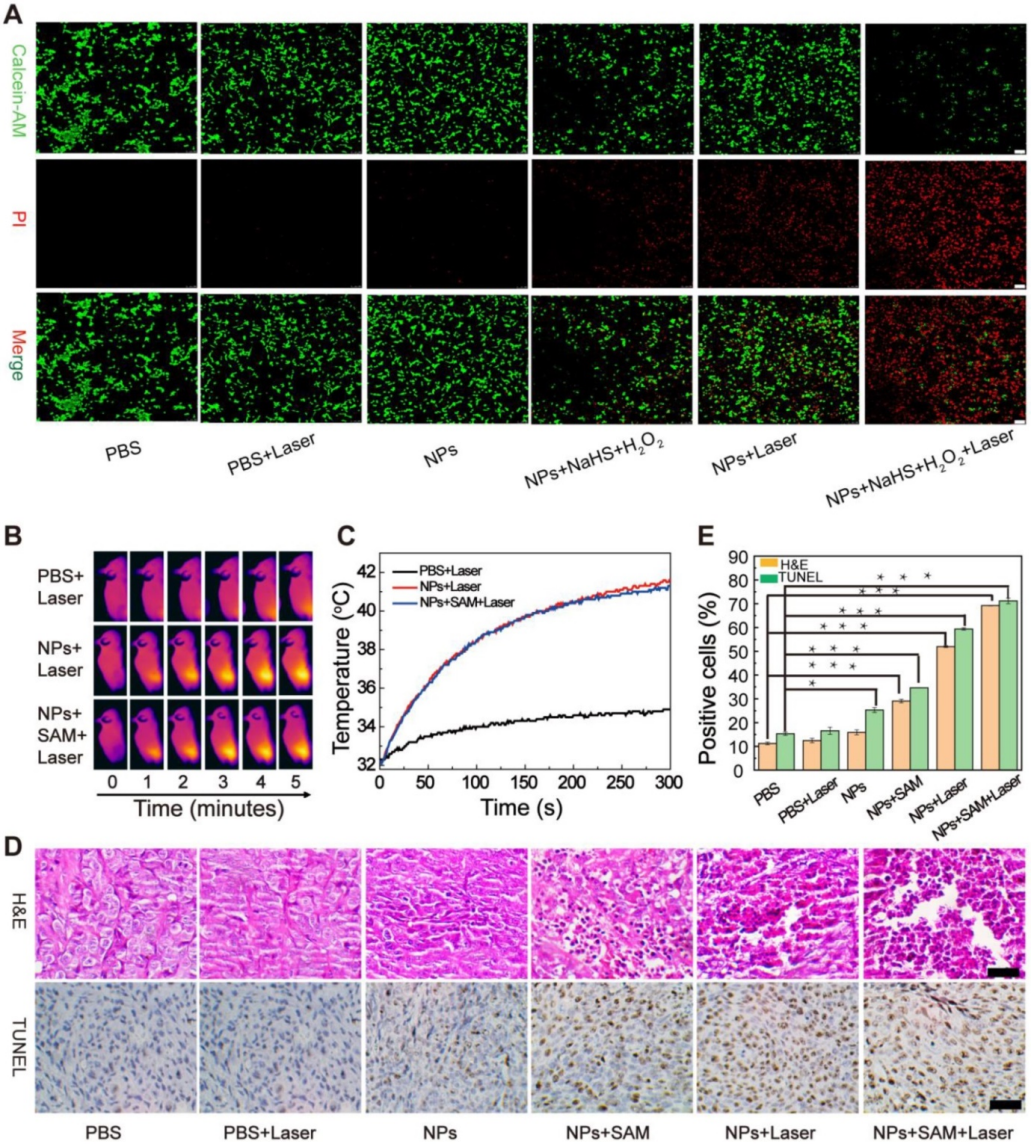
Enhanced CDT therapy using the EA-Fe@BSA NPs. (A) The Calcein-AM/PI staining of HCT116 colon cancer cells under different conditions. Scale bar = 100 μm. (B) Thermal imaging of HCT116 tumor bearing mice injected with PBS, EA-Fe@BSA NPs, or EA-Fe@BSA+SAM NPs under laser irradiation. (C) Corresponding temperature increase of the tumor site in (B). (D) H&E (top) and TUNEL (bottom) staining of tumor sections from various groups of mice (B_1_: PBS, B_2_: PBS + laser, B_3_: NPs, B_4_: NPs + SAM, B_5_: NPs + laser, and B_6_: NPs + SAM + laser). Scale bar = 50 μm. (E) Proportion of necrosis area and TUNEL-positive cells for the different groups (n = 5, ***P < 0.001, *P < 0.05).

The EA-Fe@BSA NPs displayed good enhanced CDT and PTT performance *in vitro*, their applicability to tumor treatment still needed to be confirmed *in vivo*. The enhanced CDT effect was firstly investigated in cells by Calcein-AM/PI staining (Figure 6A). HCT116 cells exhibiting time dependence on NPs uptake (Figure S19) were randomly assigned to six groups: PBS, PBS + Laser, NPs, NPs + NaHS + H_2_O_2_, NPs + Laser and NPs + NaHS + H_2_O_2_ + Laser. Obviously, the strong green fluorescence was observed in the control group (PBS, PBS + Laser), bits of dead cells were stained by PI in the NPs group. In contrast, significant green fluorescence diminished and red fluorescence intensified in the NPs + NaHS + H_2_O_2_, and NPs + Laser group, and most dead cells were appeared in NPs + NaHS + H_2_O_2_ + Laser group due to a large amount of •OH generated by the NaHS and thermal effects. The cell viability in each group are further confirmed by the MTT assay, which the NPs + NaHS + H_2_O_2_ + Laser group exhibit the lowest cell survival rate (Figure S20). Moreover, ROS-induced apoptosis process was explored by fluorescent probe 5, 5′, 6, 6′-tetrachloro-1, 1′, 3, 3′-tetraethyl-imidacarbocyanine iodide (JC-1). Obvious JC-1 monomer with green fluorescence appeared in the NPs + NaHS + H_2_O_2_ + Laser group and significant decrease of the ratio of red/green fluorescence demonstrated the depolarization of mitochondrial membrane and the occurrence of apoptosis (Figure S21 and S22) 51,52.

Inspired by this, the enhanced CDT effect were further validated* in vivo*. HCT116 tumor-bearing mice were divided into three groups (PBS, NPs, and NPs + *S*-adenosyl-L-methionine (SAM)) for intravenous injection with PBS or the NPs. The PBS group was used as the control group, and the NPs and NPs + SAM groups were used as the experimental groups. For the NPs + SAM group, the H_2_S promoter SAM which was used to promote the mice to generated more H_2_S by the body was intraperitoneally injected into the mice 24 h prior to the injection of the NPs, which was further to demonstrate that the H_2_S can accelerated Fe(III)/Fe(II) conversion to enhance the treatment effect. As the *in vivo* MRI results had revealed the highest concentration of NPs in the tumor after 5 h, the three groups were subjected to 808 nm laser irradiation 5 h after injection, and photothermal images were recorded at 0, 1, 2, 3, 4, and 5 min during irradiation (Figure 6B). For the animals in the control group, the temperature of the tumor site increased by approximately 2 °C, whereas that for the animals in the experimental groups increased by approximately 10 °C (Figure 6C), demonstrating that the EA-Fe@BSA NPs afforded a measurable photothermal heating effect in mice. It is worth noting that no difference was observed between the two experimental groups and the temperature of the tumor site for both groups increased to approximately 41 °C, which is a relatively safe temperature that could reduce the adverse effects of heat treatment on normal tissues. More importantly, H_2_S did not interfere with the PTT effect of the NPs, which is beneficial for synergistically enhancing CDT performance using PTT. Next, the influence of enhanced CDT on the tumors was evaluated at the histological level. Tissue sections of the tumor sites were excised from various groups of mice (B_1_: PBS (control), B_2_: PBS + laser (laser control), B_3_: NPs (for CDT), B_4_: NPs + SAM (for H_2_S-enhanced CDT), B_5_: NPs + laser (for PTT-enhanced CDT), and B_6_: NPs + SAM + laser (for synergistically enhanced CDT)) and subjected to hematoxylin and eosin (H&E) and terminal deoxynucleotidyl transferase dUTP nick end labeling (TUNEL) staining (Figure 6D). Almost no cellular damage was observed in the B_1_ and B_2_ groups, while a weak CDT effect was observed for the B_3_ group. In contrast, owing to the high concentration of H_2_S induced by the S-adenosyl-l-methionine (SAM) and valence conversion, the B_4_ group displayed an enhanced CDT effect in the colon tumor cells. The morphology of the tumor cells was destroyed and the nucleus was no longer in the cells. In the B_5_ group, obvious cell necrosis was observed owing to the PTT-enhanced CDT. A more prominent killing effect was observed in the B_6_ group, demonstrating outstanding enhanced CDT efficiency upon H_2_S and PTT acceleration. Quantitative analysis of the TUNEL-stained sections revealed that the proportions of TUNEL-positive cells for the B_1_, B_2_, B_3_, B_4_, B_5_, and B_6_ groups were 15.31%, 16.56%, 25.27%, 34.65%, 59.38%, and 71.18%, respectively (Figure 6E). Compared with the B_1_ and B_2_ groups, the intrinsic CDT efficiency in the B_3_ group was very low. Excitingly, a higher cell mortality rate occurred upon increasing the H_2_S concentration and in combination with PTT-promoted CDT, further validating the efficiency of the enhanced CDT approach. The above analysis revealed that the synergistically enhanced CDT group displayed the best treatment effects for colon tumors, indicating great potential for colon cancer therapy* in vivo*.

**Figure 7:**
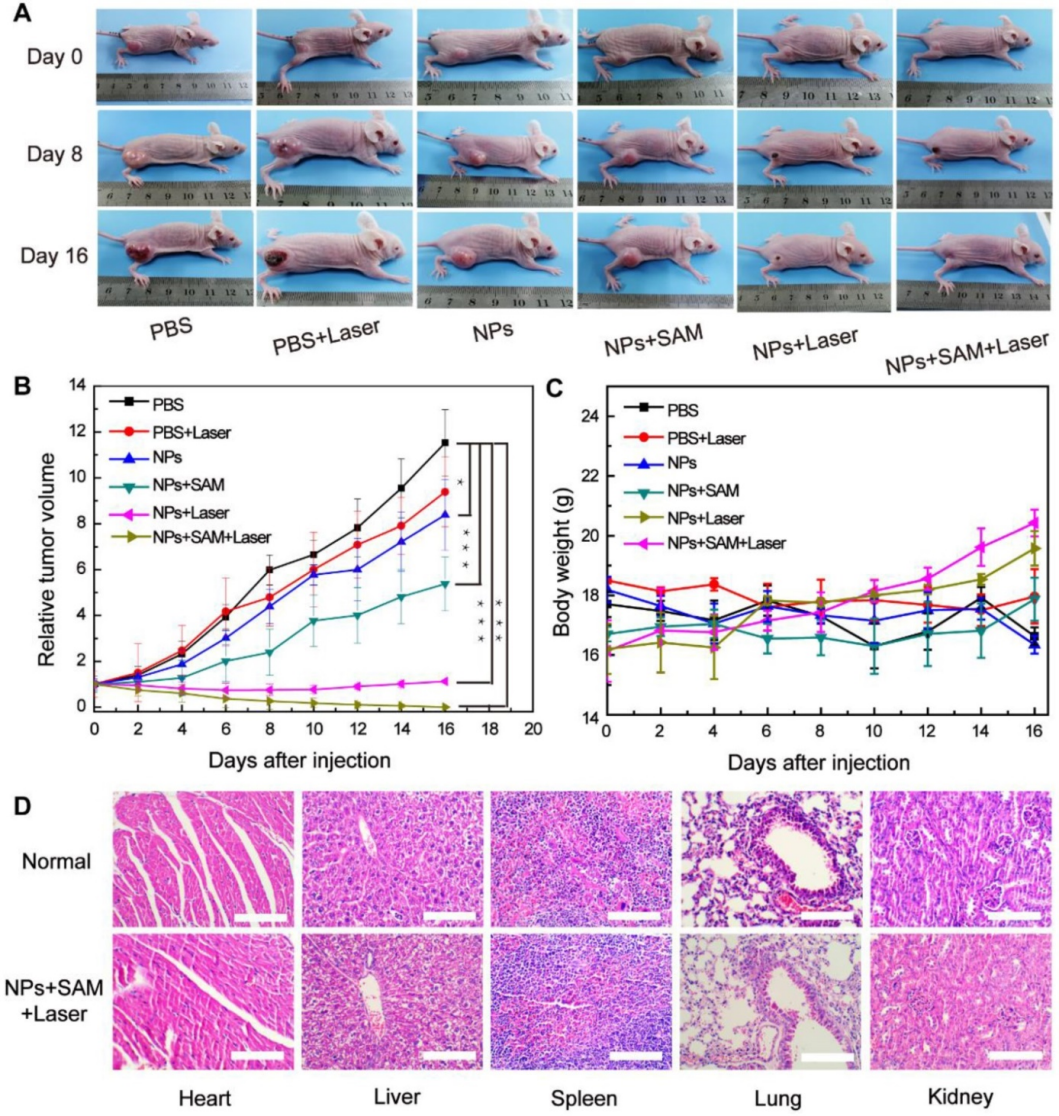
Treatment results for mice in the various groups (B_1_: PBS, B_2_: PBS + laser, B_3_: NPs, B_4_: NPs + SAM, B_5_: NPs + laser, and B_6_: NPs + SAM + laser). (A) Photographs of tumor-bearing mice on days 0, 8, and 16 of during follow-up treatment for 16 d. (B) Relative tumor volume (n = 5, ***P < 0.001, *P < 0.05). (C) Body weight. (D) H&E staining of the main organs of normal and cured mice. Scale bar = 100 μm.

The enhanced CDT effects of the EA-Fe@BSA NPs for colon tumor ablation were further explored by examining photographs of the tumor-bearing mice, tumor volume, and body weight over 16 days. As shown in Figure 7A, the tumor growth rate in the B_3_ group was lower than that in the B_1_ and B_2_ groups, which was attributed to the weak CDT effect in the B_3_ group. The tumor growth rate in the B_4_ group was also lower than that in the B_3_ group, owing to the administration of the H_2_S accelerant; more H_2_S was produced in the tumor site of the mice, leading to greater reduction of the Fe(III) in the EA-Fe@BSA NPs to Fe(II) and promoting the CDT efficiency. Unfortunately, tumor growth was still not completely inhibited under these conditions. In the B_5_ group, the tumors were inhibited in the first week, but a certain degree of recurrence occurred after 10 d in the vicinity of the original tumor. Remarkably, in the B_6_ group, the tumors were completely cured after 16 d. These results demonstrate that the synergistically enhanced CDT can effectively treat colon cancer tumors in mice. This finding was also supported by the relative tumor volume statistics for the various groups of mice. As shown in Figure 7B, the tumor growth rate increased rapidly in the B_1_, B_2_, and B_3_ groups, while the tumor volume was inhibited to a certain extent in the B_4_ group owing to the H_2_S-enhanced CDT effect of the EA-Fe@BSA NPs. Compared with the B_5_ group, the tumors in the B_6_ group were more greatly suppressed and completely cured after 16 d, demonstrating the superior tumor inhibition effect of the synergistically enhanced CDT for colon cancer treatment. Even though the H_2_S-enhanced CDT still cannot inhibit the tumor alone, the synergy of H_2_S-enhanced CDT and photothermal-enhanced CDT can remove the tumor completely. Thus, the contribution of H_2_S-enhanced CDT can further lower the requirement of laser density or irradiation time for photothermal therapy to reducing the damage to normal tissues, which can be used to guide the design of treatment plans in the future. The body weight changes for each group of mice were tracked every 2 d. As shown in Figure 7C, the body weight remained stable for all of the groups, indicating that the EA-Fe@BSA NPs and the overall treatment method induced no toxic side effects in mice. Combined with the* in vivo* treatment results, the results demonstrate that enhanced CDT and PTT alone failed to inhibit the tumors, whereas the synergistically enhanced CDT completely cured colon cancer in mice. To visually compare the therapeutic effects for the different groups, the tumors were excised after 16 d of treatment. As shown in Figure S23, the tumor size clearly decreased with the gradually enhanced CDT effects and the tumors had completely disappeared for the B_6_ group. In addition, there was no significant difference in the H&E staining sections of the main organs between the normal and cured mice (Figure 7D), which further proved the biosafety of the NPs *in vivo* and good treatment effect. All of the above data demonstrate that the CDT performance of the EA-Fe@BSA NPs was synergistically enhanced by endogenous H_2_S and PTT, and the combination of enhanced CDT and PTT based on a single agent is expected to be a more efficient therapeutic strategy for colon cancer.

## Experimental

### Materials

Iron(III) chloride hexahydrate was supplied by Sinopharm Chemical Reagent Co., Ltd. (Shanghai, China). BSA was purchased from Amresco (Solon, OH, USA). Ellagic acid (96%) was obtained from Aladdin Industrial Corporation (Shanghai, China). All reagents were used without further purification.

### Synthesis of EA-Fe@BSA NPs

BSA (66.6 mg) was dissolved in deionized water (9 mL) under magnetic stirring. Then, 108 μL of aqueous FeCl_3_ solution (0.1 g mL^-1^) was added. After stirring at room temperature for 30 min, an ethanolic solution of EA (1002 μL, 10 mg mL^-1^) was added dropwise. After stirring overnight, the solution turned dark blue. The EA-Fe@BSA NPs were collected by ultrafiltration centrifugation and washed several times with water.

### Characterization

AFM and TEM (JEM-2010F, JEOL) were used to measure the sizes and morphologies of the EA-Fe@BSA NPs. The hydrated particle size of the NPs was determined using a Malvern Zetasizer Nano ZS. The surface ion valency and composition of the NPs were measured by XPS (Axis 165, Kratos). Fluorescence spectra were recorded using a fluorescence spectrophotometer (Cary Eclipse, Agilent). NIR absorption spectra were measured using a UV-vis spectrophotometer (DU 730, Beckman Coulter). FT-IR spectra were obtained using a FT-IR spectrophotometer (Nicolet Avatar 370, Thermo).

### Biocompatibility

MTT assays were used to evaluate the toxicity of the EA-Fe@BSA NPs toward HUVECs and HCT116 colon cancer cells. First, 100 μL aliquots of cells (10^5^ mL^-1^) were plated on a 96-well plate. After incubation for 12 h, a series of RPMI 1640 medium containing the EA-Fe@BSA NPs (10, 25, 50, 100, 200 μg mL^-1^) was added to the well plate. After incubation for 12 h or 24 h, the supernatant in all wells was aspirated, MTT solution was then added to each well and allowed to stand in the incubator for another 4 h. The supernatant was then discarded, rinsed twice with PBS, followed by adding dimethyl sulfoxide (DMSO). Finally, the absorbance was measured using a microplate reader (Varioskan Flash, Thermo Fisher Scientific).

RBCs were collected from normal BALB/c nude mice (Shanghai Institutes for Biological Sciences) for hemolysis experiments to assess the biocompatibility of the NPs. First, the RBCs (0.4 mL) were added to 1 mL of water as a positive control. A second sample of RBCs was added to PBS as a negative control. Samples of RBCs were also added to PBS containing various concentrations of the NPs (25, 50, 100, or 200 μg mL^-1^). After allowing the samples to stand for 1 h, they were centrifuged at a low speed and the absorbance of the supernatant at 576 nm was measured using a UV-vis spectrophotometer. The percent hemolysis was then calculated using the formula:

(*A*_t_-*A*_nc_)/(*A*_pc_-*A*_nc_)×100%,

where *A*_pc_, *A*_t_, and *A*_nc_ represent the absorbances of the positive control, NP sample, and negative control at 576 nm, respectively.

Routine blood experiments were performed to further assess the toxicity of the EA-Fe@BSA NPs to mice. Blood samples were collected from three normal BALB/c nude mice and three treated mice for routine blood analysis.

### *In Vitro* and *In Vivo* MRI Performance

The *in vitro* transverse relaxation rate, longitudinal relaxation rate, and MRI were measured on a 0.5 T MRI instrument, and various Fe concentrations of EA-Fe@BSA NPs (0.05, 0.10, 0.20, 0.40, 0.80, and 1.60 mM) were added to centrifuge tubes for MRI scanning. The* in vitro T*_1_-weighted MRI parameters included an echo time (TE) of 0.04 ms, a spectrometer frequency offset of the first channel (SFO1) of 18.538 MHz, a repetition time (TR) of 200 ms, a slice width of 4 mm.

*In vivo T*_1_-weighted MRI was conducted on a 0.5 T MRI instrument (MiniMR-60, Niumag), BALB/c nude mice inoculated with HCT116 cells (Shanghai Experimental Animal Center) were used in the experiments. Coronal scanning was performed before and after the injection of the EA-Fe@BSA NPs (1, 3, 5, 6, and 7 h). The dosage of EA-Fe@BSA NPs was 100 μL (3.5 mM). The *in vivo T*_1_-weighted MRI parameters included a field of view of 80 × 80 mm, a TR of 340 ms, a TE of 18.125 ms, a slice thickness of 3 mm.

### *In Vitro* and *In Vivo* Photothermal Performance

To evaluate the photothermal performance *in vitro*, a thermal imaging camera (A300, FLIR) was used to measure the temperature change for pure water and various concentrations of the EA-Fe@BSA NPs. To evaluate the photothermal stability of the NPs, the sample was subjected to laser irradiation (808 nm laser, 1 W cm^-2^) for 15 min followed by a cooling down period of 15 min, and this cycle was repeated six times. The photothermal conversion efficiency was calculated according to our previous report 53.

To evaluate the photothermal performance *in vivo*, 100 μL (3.5 mM) of the NPs or PBS were separately injected into HCT116 tumor bearing mice through the tail vein. After 5 h, the tumor sites were irradiated with an 808 nm laser (1 W cm^-2^) for 5 min and the temperature change was recorded by thermography.

### Enhanced CDT Performance* In Vitro*

The CDT performance was assessed using the levels of •OH. The MB colorimetric method was used to monitor the generation of •OH under various conditions, and H_2_S was simulated using NaHS. The experiment involved seven samples (A_1_: PBS, A_2_: H_2_O_2_, A_3_: NPs, A_4_: NPs + NaHS, A_5_: NPs + H_2_O_2_, A_6_: NPs + NaHS + H_2_O_2_ at 298 K, and A_7_: NPs + NaHS + H_2_O_2_ at 313 K). The concentrations of the EA-Fe@BSA NPs, NaHS, and H_2_O_2_ were 0.2 mM, 3 mM, and 100 μM, respectively. A_1_-A_6_ samples were stored at 298 K for 30 min and A_7_ samples were stored at 313 K for 30 min, then subjected to ultrafiltration centrifugation; the NPs were left in the ultrafiltration tube following centrifugation and absorbance of the filtrate was measured.

The •OH produced in the different samples was further measured via X-band ESR spectroscopy using DMPO as a capture agent for •OH. DMPO solution (100 μL, 100 mM) was added to each sample and the resulting mixture was transferred to a quartz capillary for measurement of the ESR spectra (E500-10/12, Bruker).

To evaluate the CDT performance in cells, DCFH-DA was used as a fluorescence indicator and the intracellular ROS production was measured via confocal laser scanning imaging. First, HCT116 cells seeded in specific culture dishes were incubated at 37 °C for 12 h under a 5% CO_2_ atmosphere. Next, they were incubated with the various samples (A_1_-A_7_; 100 μg/mL NPs, 3 mM NaHS, and 100 μM H_2_O_2_) for 5 h, washed twice with PBS, and then dyed with DCFH-DA (10 μM) for 30 min. After washing with PBS, the fluorescence was detected via confocal laser scanning microscopy (TCS SP5, Leica, Germany).

For Calcein-AM/PI staining, HCT116 cells were seeded in glass based dishes and incubated at 37 °C for 12 h under a 5% CO_2_ atmosphere. Next, they were incubated with the various samples (PBS, PBS + Laser, NPs, NPs + NaHS + H_2_O_2_, NPs + Laser, NPs + NaHS + H_2_O_2_ + Laser; 100 μg/mL NPs, 3 mM NaHS, and 100 μM H_2_O_2_) for 5 h. For groups requiring laser irradiation, the cells were irradiated by 808 nm laser for 5 min (1 W/cm^2^), and then dyed with calcein-AM and PI solution for 25 min. After washing with PBS, the fluorescence was detected via confocal laser scanning microscopy.

### Enhanced CDT Performance *In Vivo*

HCT116 tumor bearing mice (enrich in H_2_S) were divided into six groups (B_1_: PBS, B_2_: PBS + laser, B_3_: NPs, B_4_: NPs + SAM, B_5_: NPs + laser, and B_6_: NPs + SAM + laser) with six mice per group. SAM is a common inducer that can improve CBS enzymatic activity, leading to increased H_2_S concentrations. First, the animals in the B_4_ and B_6_ groups were injected with SAM, and 24 h later the animals in the B_1_ and B_2_ groups were injected with 100 μL PBS while those in the B_3_, B_4_, B_5_, and B_6_ groups were injected with 100 μL (3.5 mM) Fe concentrations of NPs. After a further 5 h, the animals in the B_2_, B_5_, and B_6_ groups were subjected to irradiation with an 808 nm laser (1 W cm^-2^) for 5 min. Subsequently, the tumors were excised from one mouse in each group for H&E and TUNEL staining. Finally, the body weight and tumor volume (tumor width^2^ × tumor length/2) of the remaining mice in each group were monitored for 16d.

## Conclusion

In summary, ultra-small EA-Fe@BSA NPs with good biocompatibility were synthesized via the simple assembly of a natural polyphenol, Fe(III) and albumin for enhanced CDT.* In vitro* enhanced CDT results revealed that a significant amount of •OH was detected upon the addition of NaHS and increasing the temperature. The Fe(III) with low catalytic activity was rapidly reduced to Fe(II) by the abundant H_2_S, providing a rapid Fe(III)/Fe(II) conversion system to improve the regeneration of Fe(II). Importantly, the EA-Fe@BSA NPs exhibited strong NIR absorption and excellent photothermal conversion efficiency both *in vitro* and *in vivo*, which is beneficial for tumor inhibition by PTT-enhanced CDT. Moreover, the low *r*_2_:*r*_1_ ratio (1.05) and good MRI performance of the as-obtained EA-Fe@BSA NPs indicated great promise as a *T*_1_-weighted MRI diagnostic reagent. Tumor ablation experiment results demonstrated that endogenous H_2_S and PTT could synergistically enhance the CDT efficiency, significantly suppressing and curing tumors in mice. After injection of the EA-Fe@BSA NPs into mice and their transportation to the unique microenvironment of colon cancer tumors, the NPs not only underwent Fe(III) reduction to Fe(II) by endogenous H_2_S, thereby expediting the Fe(III)/Fe(II) transformation for CDT enhancement based on the Fenton reaction, but also mediated PTT effects under NIR irradiation to generate more •OH, thereby realizing synergistically enhanced CDT. This synergistically enhanced CDT strategy, which exploits endogenous reducing substances in tumor cells, provides a promising paradigm for colon cancer treatment.

## Supplementary Material

Supplementary figures.

## Abbreviations

CDT: chemodynamic therapy
H_2_S: endogenous hydrogen sulfide
EA-Fe@BSA: ellagic acid-Fe-bovine serum albumin
NIR: near-infrared
•OH: hydroxyl radicals
MRI: magnetic resonance imaging
CBS: cystathionine-β-synthase
PTT: photothermal therapy
ROS: reactive oxygen species
AFM: atomic force microscopy
TEM: transmission electron microscopy
FT-IR: fourier-transform infrared
XPS: X-ray photoelectron spectroscopy
NaHS: Sodium hydrosulfide
ESR: electron spin resonance
DMPO: 5,5-Dimethyl-1-pyrroline *N*-oxide
DCFH-DA: 2ʹ,7ʹ-dichlorodihydrofluorescein diacetate
HUVECs: human umbilical vein endothelial cells
H&E: hematoxylin and eosin
TUNEL: terminal deoxynucleotidyl transferase dUTP nick end labeling
SAM: S-adenosyl-l-methionine
