# Supplementary material for: Ellagic acid-Fe@BSA nanoparticles for endogenous H2S accelerated Fe(III)/Fe(II) conversion and photothermal synergistically enhanced chemodynamic therapy
Source: Theranostics. 2020 Mar 4;10(9):4101–15. doi: 10.7150/thno.41882 (PMC7086347; doi:10.7150/thno.41882)
Supplement: Supplementary file 1 — Supplementary figures. [file thnov10p4101s1.pdf]

Ellagic Acid–Fe@BSA Nanoparticles for Endogenous H_2S Accelerated Fe(III)/Fe(II) Conversion and Photothermal Synergistically Enhanced Chemodynamic Therapy

Qingqing Tian, Lu An, Qiwei Tian*, Jiaomin Lin, Shiping Yang*

The Key Laboratory of Resource Chemistry of the Ministry of Education, the Shanghai Key Laboratory of Rare Earth Functional Materials, and the Shanghai Municipal Education Committee Key Laboratory of Molecular Imaging Probes and Sensors, Shanghai Normal University, Shanghai, 200234 (China)

*E-mail: anlu1987@shnu.edu.cn, qiweitian@shnu.edu.cn

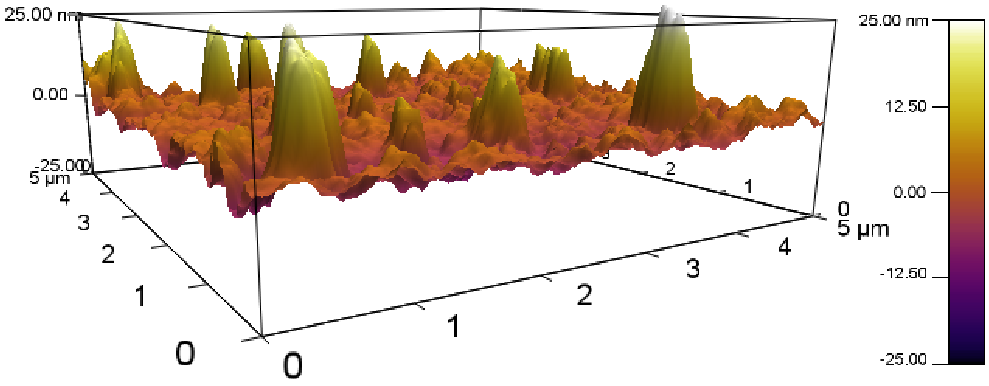

Figure S1. 3D AFM topography of the EA-Fe@BSA NPs.

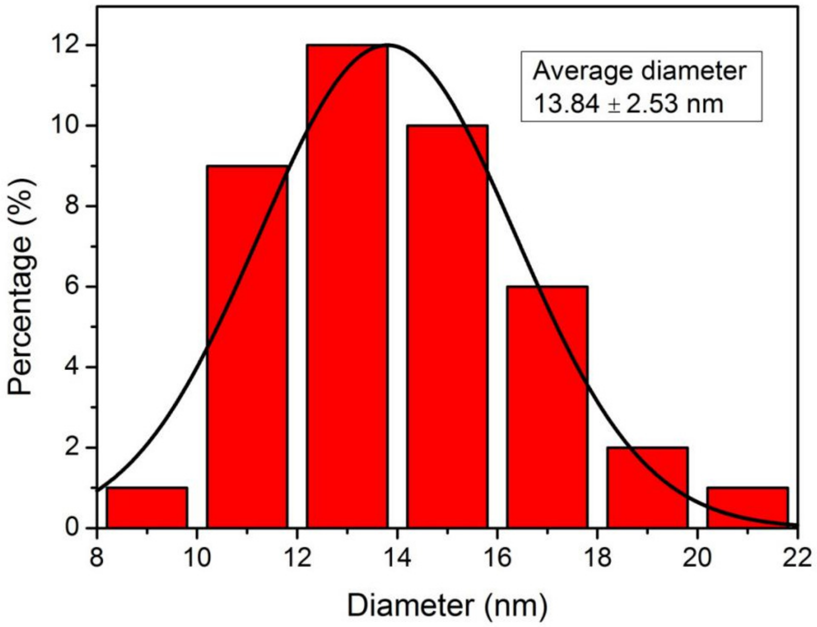

Figure S2. Particle size distribution of the EA-Fe@BSA NPs based on TEM.

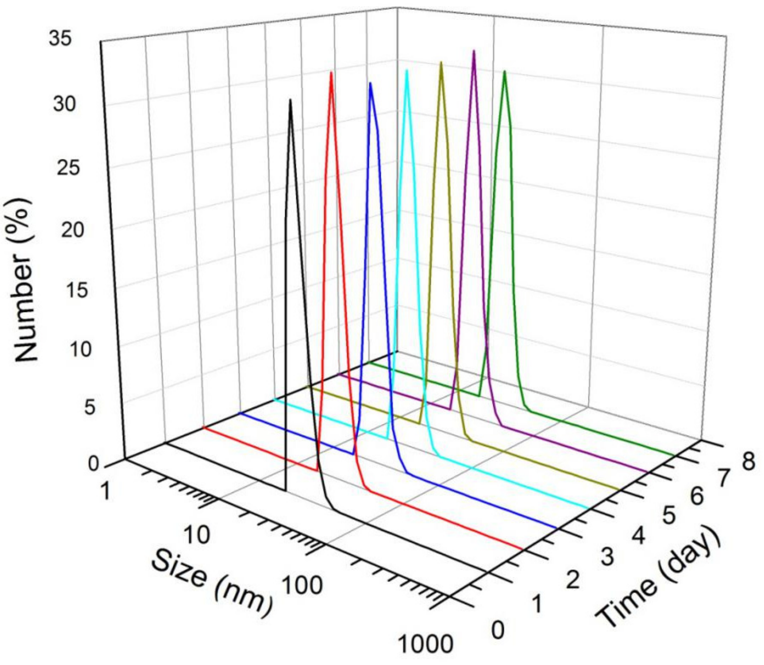

Figure S3. Size stability of as-synthesized EA-Fe@BSA NPs.

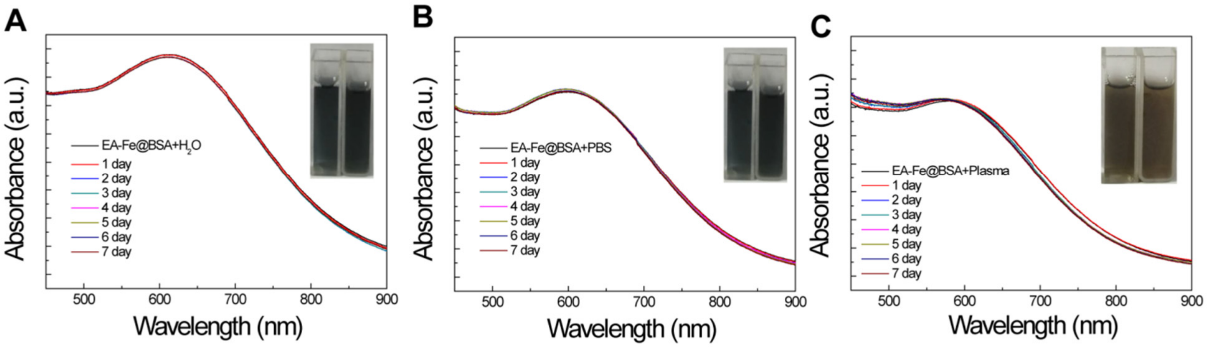

Figure S4. The stability of EA-Fe@BSA NPs in different physiological solution for one week. Absorption spectrum collected every day for one week and corresponding photograph (inset) collected at 1 day and 7 day of the EA-Fe@BSA NPs. (A-C) dispersed in H_2O, PBS and plasma, respectively.

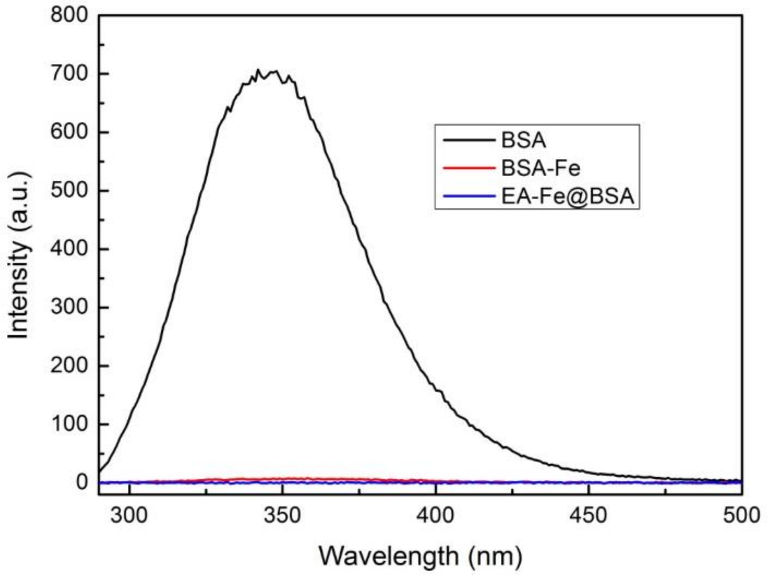

Figure S5. Fluorescence spectra of pure BSA, BSA-Fe, and the EA-Fe@BSA NPs.

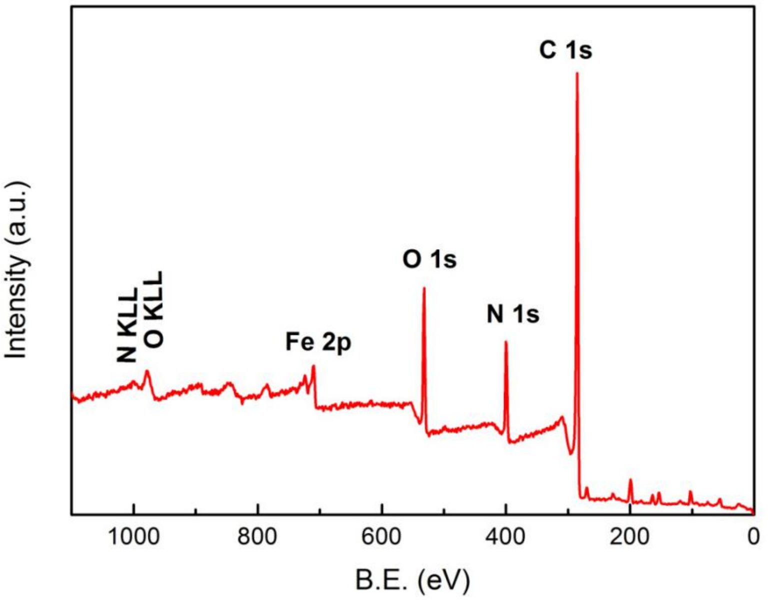

Figure S6. XPS spectra of the EA-Fe@BSA NPs.

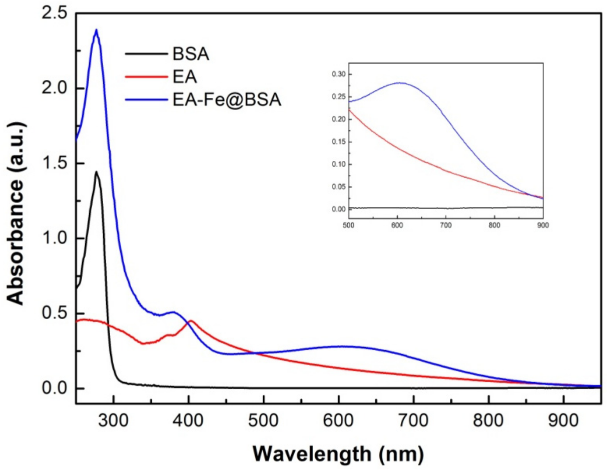

Figure S7. The absorbance of BSA, EA and EA-Fe@BSA.

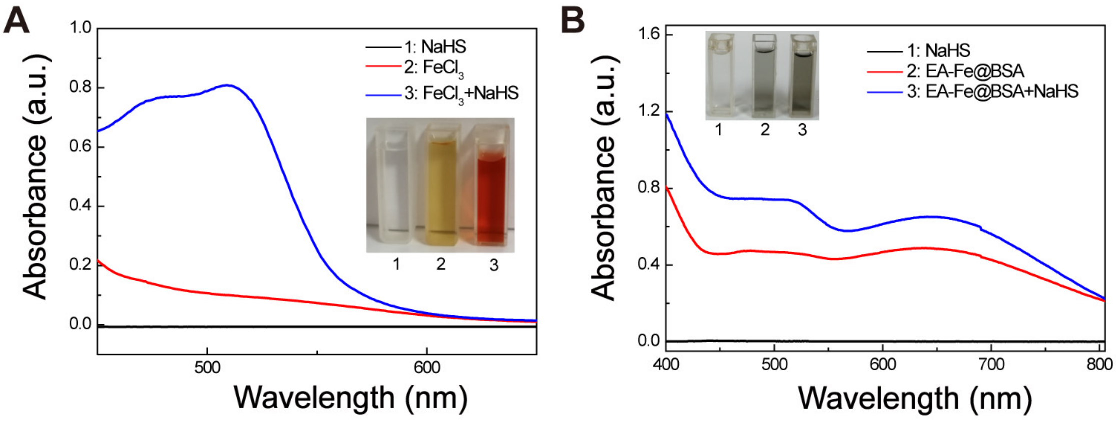

Figure S8. Detection of Fe^{2+} concentration in the dark: UV spectra of 1,10-phenanthroline in the presence of NaHS, $FeCl_3$, and $FeCl_3$ + NaHS. The absorption peak at 511 nm corresponds to the complex between 1,10-phenanthroline and Fe^{2+} ions. [$FeCl_3$] = 1.0 mg/mL, [NaHS] = 3 mM, [phenanthroline] = 0.5 mg/mL. (A) Detection of Fe^{2+} concentration in the EA-Fe@BSA NPs: UV spectra of 1,10-phenanthroline in the presence of NaHS, EA-Fe@BSA NPs, and EA-Fe@BSA NPs + NaHS. The absorption peak at 511 nm corresponds to the complex between 1,10-phenanthroline and Fe^{2+} ions. (B)

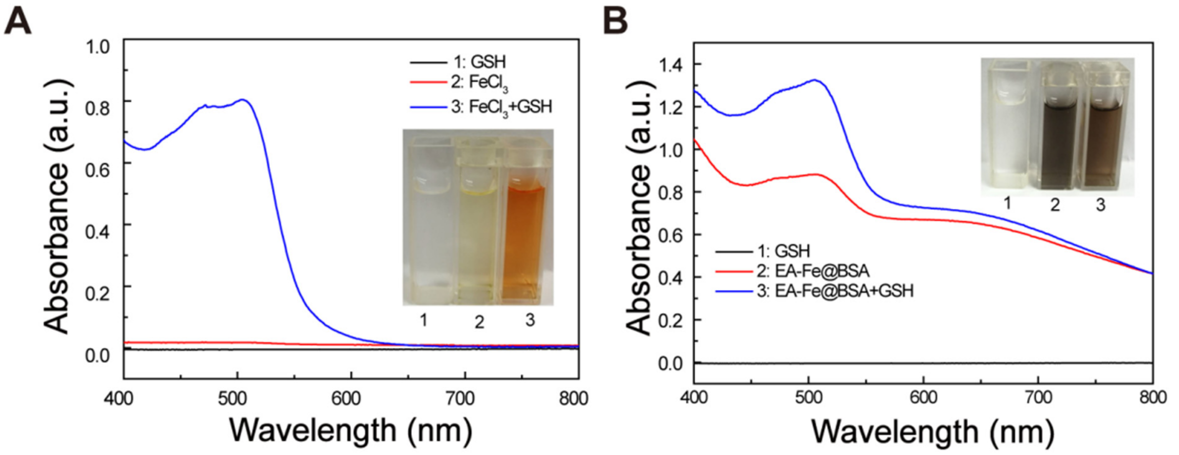

Figure S9. Detection of Fe^{2+} concentration in the dark: UV spectra of 1,10-phenanthroline in the presence of GSH, $FeCl_3$, and $FeCl_3$ + GSH. The absorption peak at 511 nm corresponds to the complex between 1, 10-phenanthroline and Fe^{2+} ions. (A) Detection of Fe^{2+} concentration in the EA-Fe@BSA NPs: UV spectra of 1, 10-phenanthroline in the presence of GSH, EA-Fe@BSA NPs, and EA-Fe@BSA NPs + GSH. The absorption peak at 511 nm corresponds to the complex between 1,10-phenanthroline and Fe^{2+} ions. (B)

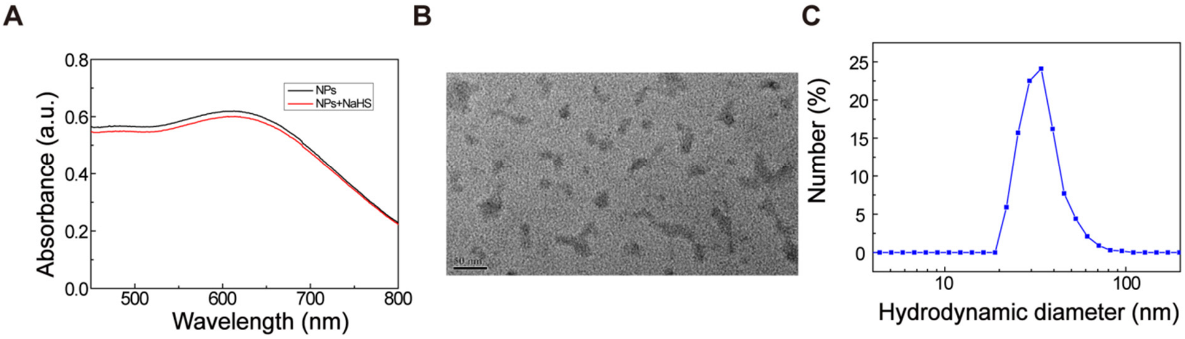

Figure S10. Characterization of the EA-Fe@BSA + NaHS: (A) absorbance spectrum, (B) TEM image, (C) hydrodynamic diameter profile.

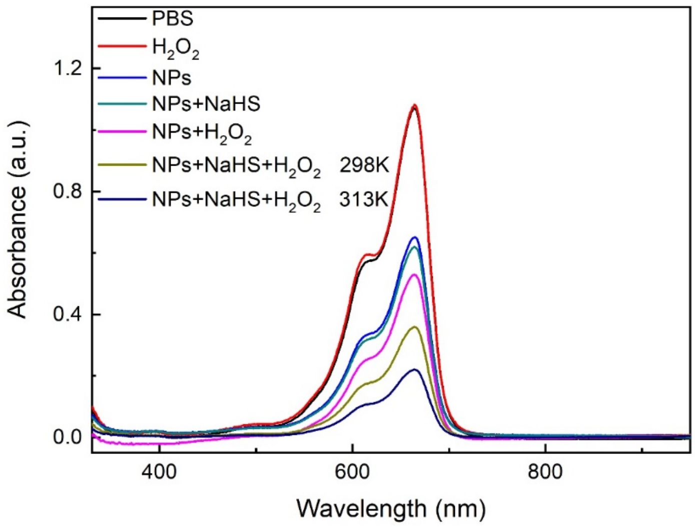

Figure S11. Absorbance of MB under various conditions (PBS, H_2O_2, NPs, NPs + NaHS, NPs + H_2O_2, NPs + NaHS + H_2O_2 at 298 K, and NPs + NaHS + H_2O_2 at 313 K).

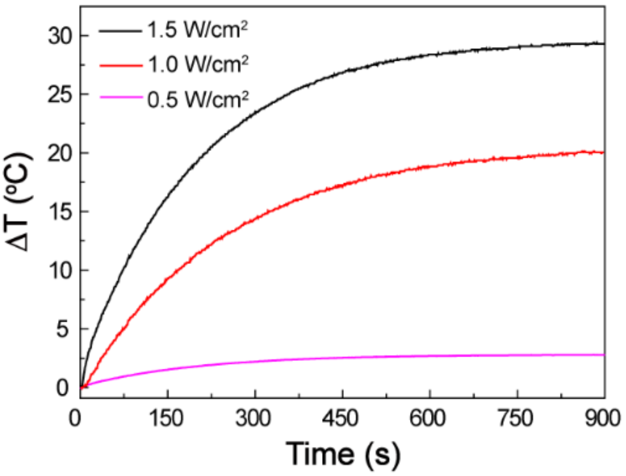

Figure S12. Photothermal heating curves of the EA-Fe@BSA NPs at Fe concentrations (0.80 mM) under irradiation with an 808 nm laser (1.5 W/cm^2, 1 W/cm^2, 0.5 W /cm^2).

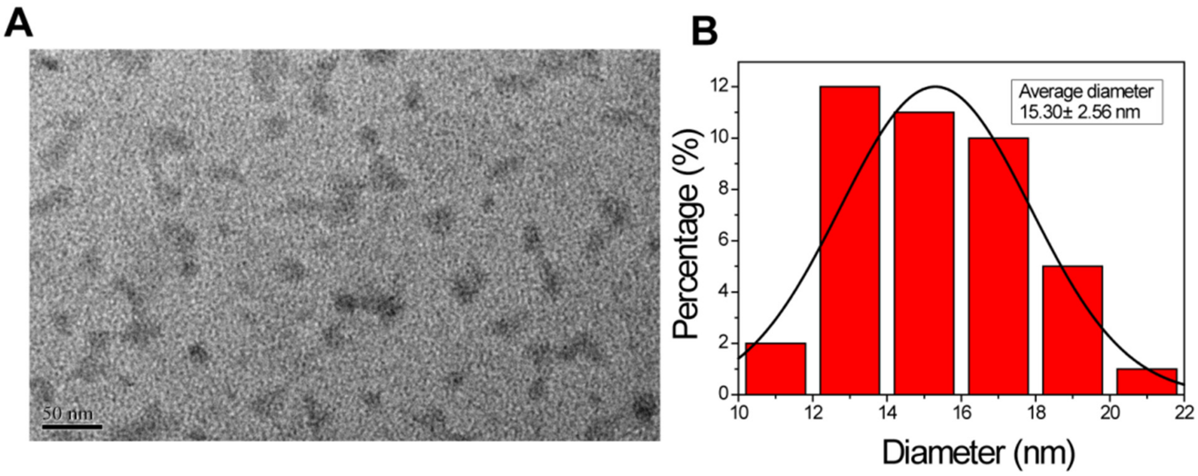

Figure S13. TEM and particle size distribution of EA-Fe @ BSA NPs after light irradiation based on TEM.

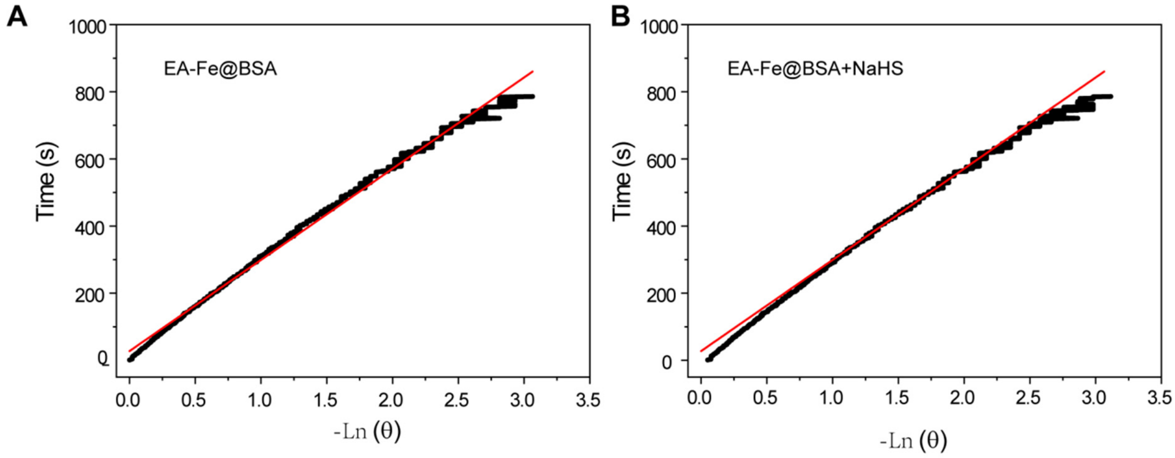

Figure S14. Linear fitting curve of time data versus $-\ln\theta$ obtained from the cooling period of the EA-Fe@BSA NPs before (A) and (B) after adding NaHS.

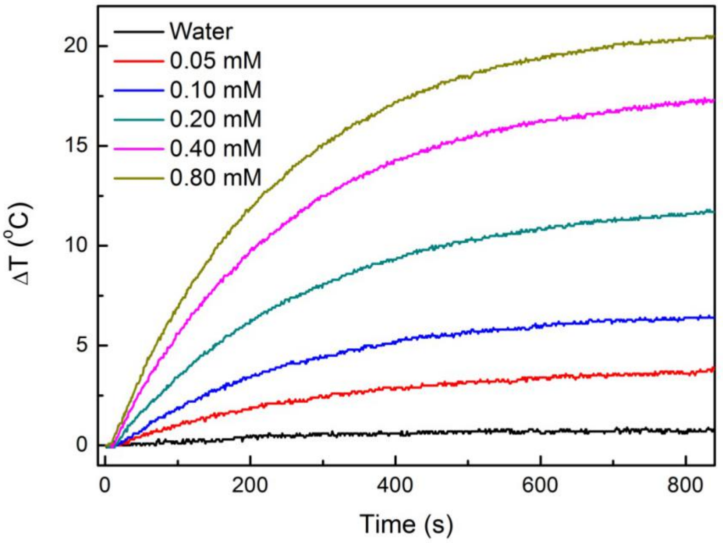

Figure S15. Photothermal curves for EA-Fe@BSA NPs + NaHS at various Fe concentrations (0, 0.05, 0.10, 0.20, 0.40, and 0.80 mM) under irradiation with an 808 nm laser (1 W cm^{-2}).

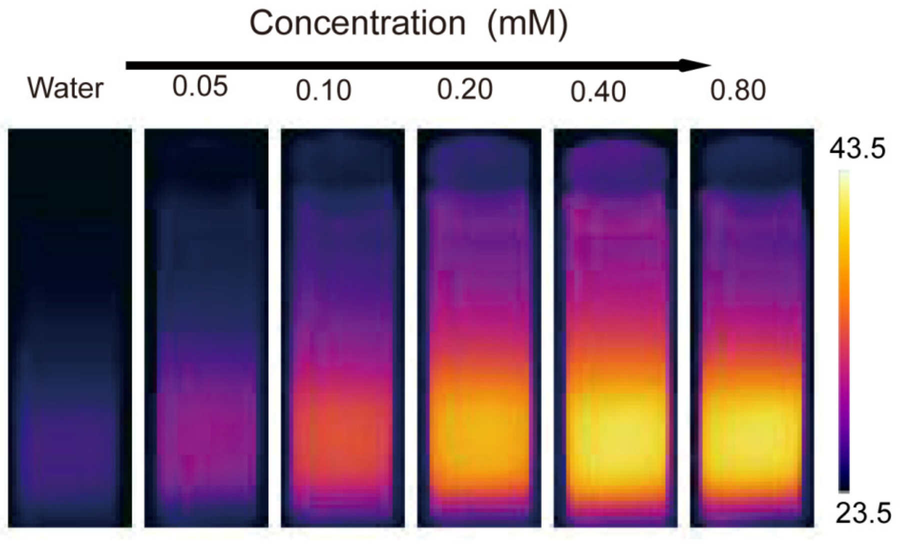

Figure S16. NIR thermal images of the EA-Fe@BSA NPs + NaHS.

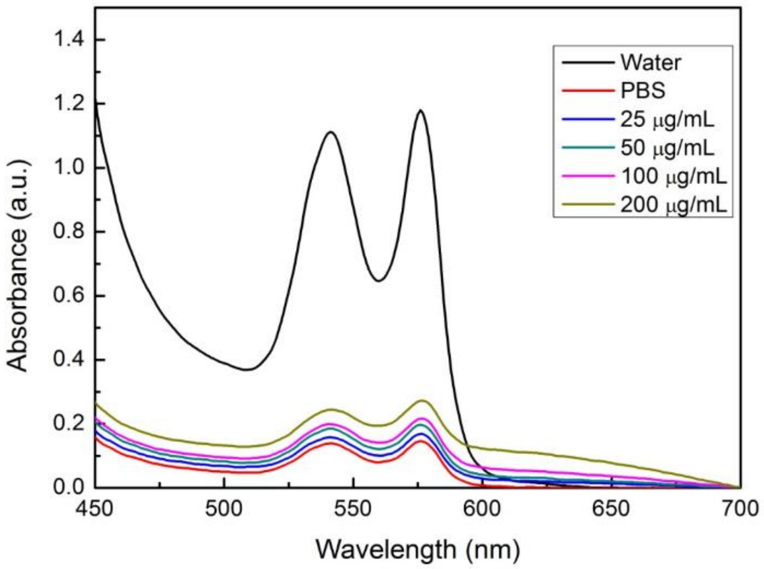

Figure S17. Absorbance of supernatants at various concentration in the hemolysis assay.

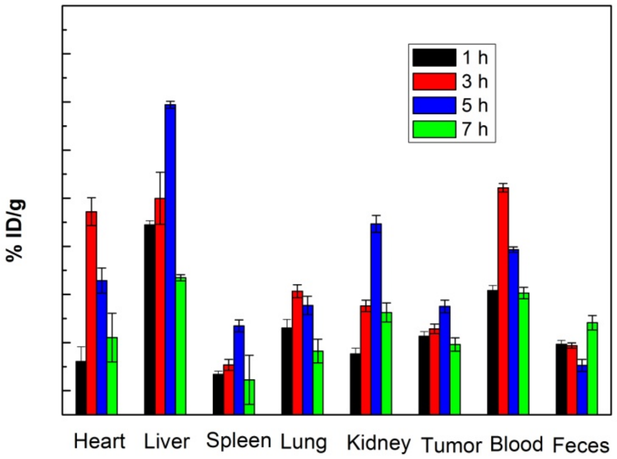

Figure S18. Biodistribution of EA-Fe@BSA NPs in main organs, tumors, blood and feces of the mice after 1, 3, 5 and 7 h of intravenous injection of EA-Fe@BSA NPs. %ID/g = Fe content in organ and tissue /injection dose of Fe/organ and tissue weight × 100%.

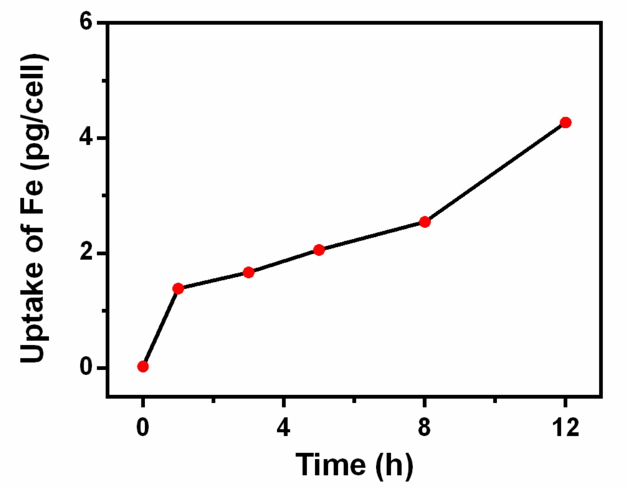

Figure S19. Time-dependent cellular uptake of the EA-Fe@BSA NPs by HCT116 cells at Fe concentration of 0.8 mM (45 μg/mL).

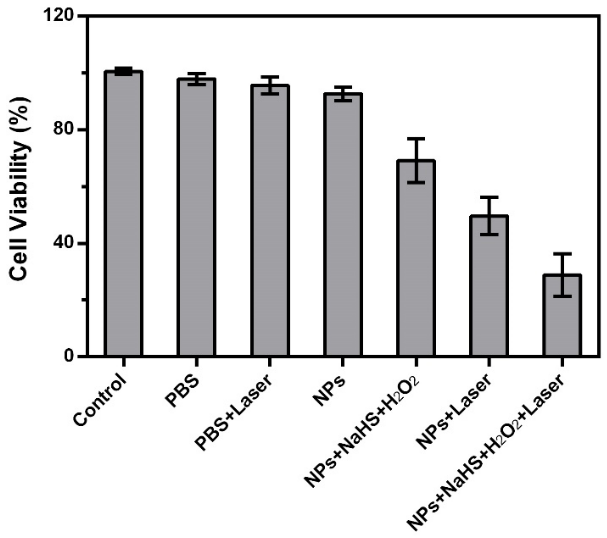

Figure S20. *In vitro* enhanced CDT therapy measured by MTT assay.

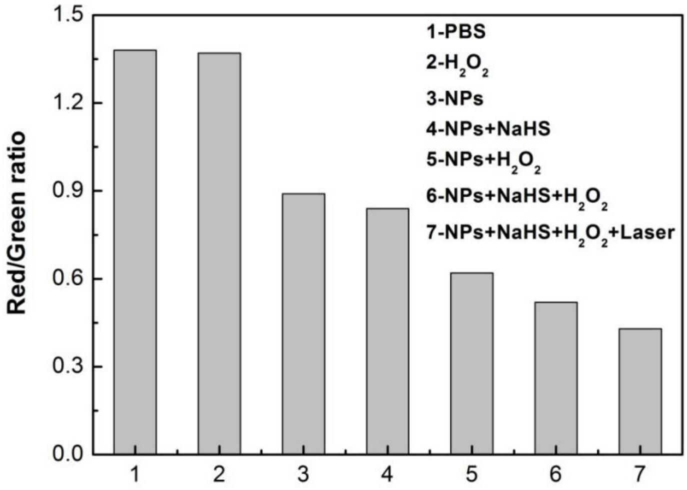

Figure S21. Flow cytometry chart to detect cell mitochondrial membrane potential.

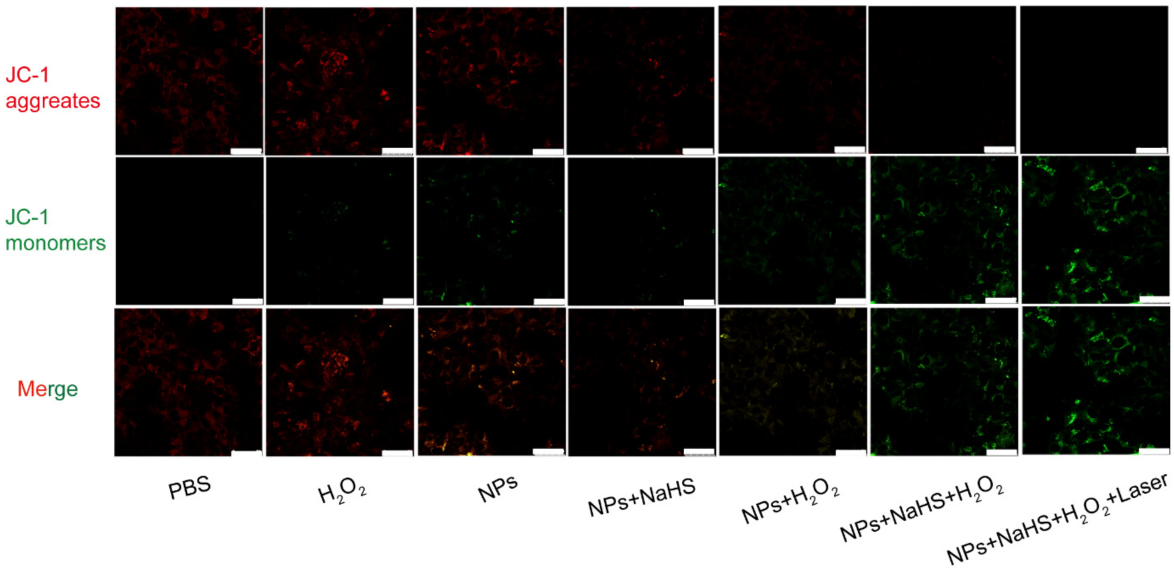

Figure S22. Confocal laser scanning microscopy images of HCT116 colon cancer cells stained with JC-1 under various conditions (A_1: PBS, A_2: H_2O_2, A_3: NPs, A_4: NPs + NaHS, A_5: NPs + H_2O_2, A_6: NPs + NaHS + H_2O_2, and A_7: NPs + NaHS + H_2O_2 +Laser; scale bar = 50 µm).

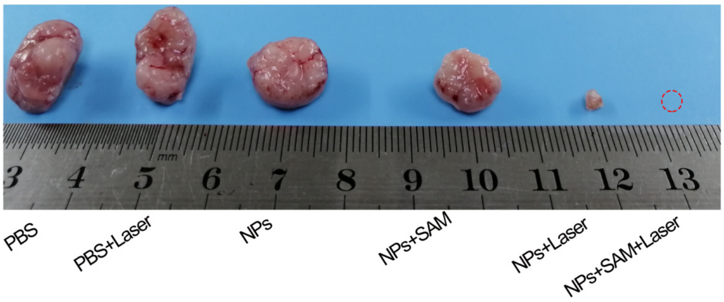

Figure S23. Tumor photographs of tumor mice in different groups after 16 days of therapy (PBS, PBS + laser, NPs, NPs + SAM, NPs + laser, and NPs + SAM + laser).
